# Application of Large Language Models in Chronic Disease Care: Mixed Methods Systematic Review and Thematic Synthesis

**DOI:** 10.2196/90744

**Published:** 2026-08-11

**Authors:** Linghui Zhang, Panpan Huai, Rui Xu, Jingjing Sun, Ruihua Jin, Huimei Lv

**Affiliations:** 1 Shanxi Medical University, Taiyuan, Shanxi, China; 2 The Fifth Clinical College of Shanxi Medical University, No. 29, Shuangta Temple Street, Yingze District, Taiyuan, Shanxi, 030012, China, 86 13073585857

**Keywords:** large language models, chronic disease care, mixed methods systematic review, thematic synthesis, Orem's Self-Care Theory

## Abstract

**Background:**

Chronic diseases account for nearly three-quarters of global deaths and demand continuous, personalized long-term management; yet traditional care models often fall short in delivering such sustained support. Large language models, with advanced conversational and analytical capabilities, present promising opportunities to address the problem by offering scalable, interactive support. However, a comprehensive synthesis of evidence across diverse study designs, which moves beyond isolated technical metrics to evaluate large language models through a structured, theory-driven lens, remains limited.

**Objective:**

This study aimed to synthesize quantitative, qualitative, and mixed methods evidence on technical performance, application scenarios, and documented challenges of large language models in chronic disease care, and to critically evaluate these findings through a theory-driven, 3D framework informed by Orem’s Self-Care Theory.

**Methods:**

The mixed methods systematic review adhered to the PRISMA (Preferred Reporting Items for Systematic Reviews and Meta-Analyses) 2020 and SWiM (Synthesis Without Meta-Analysis) guidelines. A comprehensive computer-based search was conducted across PubMed, Web of Science, Embase, Cochrane Library, CINAHL, Wiley Online Library, SpringerLink, ScienceDirect, China National Knowledge Infrastructure, Wanfang Data, VIP Database, and the China Biology Medicine Disc from inception to April 2026. Two independent researchers performed study screening, data extraction, and quality appraisal using the Mixed Methods Appraisal Tool 2018. Given significant clinical and methodological heterogeneity across the included studies, a quantitative meta-analysis was not appropriate; instead, thematic synthesis was used following Thomas and Harden’s 3-step approach, with NVivo 14 (Lumivero) used for line-by-line coding and theme development, all informed by Orem’s 3D framework.

**Results:**

A total of 20 studies were included, all rated as moderate or high quality. The thematic synthesis revealed three core themes aligned with the proposed framework: (1) foundational safety, privacy, and fairness (hallucination risks and data concerns); (2) self-care enablement through perceived usefulness (patient education, decision support, and self-management); and (3) design and system integration challenges (readability mismatches and workflow gaps). Patient education and clinical decision support were the most common application scenarios. Key technical enhancements (retrieval-augmented generation [RAG] and fine-tuning) primarily strengthened the second theme, while barriers such as content readability, hallucination risks, and ethical ambiguities limited real-world readiness.

**Conclusions:**

This review is the first to integrate Orem’s Self-Care Theory into a 3D evaluative framework for large language models in chronic care, thereby moving beyond fragmented, technology-centric assessments toward a structured, nursing-informed, and theory-driven synthesis. Unlike prior reviews that primarily focused on isolated technical metrics or broad feasibility, this synthesis provides a layered, discipline-grounded evaluation distinguishing foundational safety, self-care enablement, and system integration. These findings show evidence mainly supports the intermediate layer, while safeguards and operational integration remain deficient, guiding nursing research toward safety assurances, health-literacy-adaptive design, and implementation science for equitable, patient-centered care.

## Introduction

### Background

Chronic noncommunicable diseases refer to conditions that are not primarily caused by acute infections, characterized by long duration and typically slow progression, and requiring long-term treatment and care [1]. Major categories include cardiovascular diseases (eg, hypertension, coronary heart disease, and stroke), cancers, chronic respiratory diseases (eg, chronic obstructive pulmonary disease and asthma), diabetes mellitus, chronic kidney disease, digestive system diseases (eg, chronic hepatitis, cirrhosis, and inflammatory bowel disease), musculoskeletal disorders (eg, arthritis and fibromyalgia), and neurodegenerative conditions (eg, Parkinson disease and Alzheimer disease) [2]. According to World Health Organization statistics, chronic diseases account for nearly three-quarters of all deaths globally, with approximately 17 million people dying from a chronic disease before the age of 70 each year, 86% of which occur in low- and middle-income countries [1,3]. The Lancet has noted that the current burden of healthy life-years lost and premature mortality due to chronic diseases remains substantial [4]. With accelerating population aging and epidemiological transition, chronic disease care faces multiple challenges, including a massive patient population, diverse health needs, and prolonged management cycles [5]. Traditional chronic disease care models, largely dependent on regular outpatient follow-ups and standardized health education, struggle to deliver continuous, personalized, and dynamic interventions [6]. This limitation is particularly pronounced in regions with unevenly distributed medical resources and a shortage of specialized professionals [7]. Among major chronic diseases, conditions such as diabetes, hypertension, and nonalcoholic fatty liver disease affect tens of millions to hundreds of millions of people globally [8,9]. Conditions like hypertension and diabetes demand high continuity and personalization in care due to the necessity for ongoing monitoring, behavioral interventions, and frequent health information needs [10].

The World Health Organization’s 14th General Program of Work (2025‐2028) emphasizes leveraging emerging technologies like AI and strengthening digital interventions to support health literacy [11]. Integrating this model with precision care has become a significant trend [12]. Currently, information management platforms such as mobile health apps and smart wearable devices have shown initial success in chronic disease data collection and remote monitoring but still possess limitations [13]. Research by Cunningham et al [14] found that while common smart follow-up tools can perform basic data collection, their information integration capabilities are limited, making it difficult to extract personalized insights from diverse health data. Large language models (LLMs), as a groundbreaking AI technology, leverage powerful natural language understanding and generation capabilities and have rapidly developed in the health care domain [15]. LLM platforms such as OpenAI’s ChatGPT, Google’s Gemini, Anthropic’s Claude, and DeepSeek have demonstrated intelligent support capabilities in nursing scenarios [16]. The application of LLMs has permeated various health care fields, showing significant value in education and clinical practice [17-19]. Research by Harrington et al [20] points out that LLMs can act as intelligent tutors in nursing education. Studies have also found that LLMs can serve as 24/7 information assistants, responding to general inquiries about disease symptoms, medications, and lifestyle [21]. Patients can ask questions conversationally about diet control, exercise safety, or medication side effects and receive detailed explanations [22]. Compared to the preset, static responses of traditional clinical decision support systems, this flexible, interactive method demonstrates stronger adaptability [23]. The characteristics of chronic disease care—long duration, need for continuous health education, and daily support—align well with the advantages LLMs offer [24].

However, alongside these promising capabilities, significant concerns have emerged. Studies have consistently reported that LLMs may exhibit “hallucinations”—generating inaccurate or even dangerous medical information [25,26]. In the context of chronic disease management, where patients often have complex comorbidities and rely on precise medication and dietary advice, such errors could lead to adverse health outcomes [27]. Furthermore, LLMs may misinterpret diverse, colloquial patient descriptions, particularly from individuals with lower health literacy or nonstandard language use [28]. Additional barriers include data privacy and security risks, unclear ethical and legal responsibility for AI-generated recommendations, and potential algorithmic biases that could disadvantage certain patient populations [29]. Moreover, the readability of LLM-generated content often exceeds the comprehension level of average patients, limiting its practical utility for self-care [30,31]. Given this duality, substantial potential alongside considerable risks—a systematic and critical synthesis of the evidence is urgently needed [32].

Li et al [33] conducted a mixed method review of LLMs in chronic disease management, with a primary focus on quantitative efficacy indicators. Watson et al [34] performed an integrative review on generative AI in general nursing practice but did not specifically address chronic disease care. Existing reviews on LLMs in chronic care have primarily focused on technical performance metrics (eg, accuracy and precision) or have broadly cataloged application scenarios without a structured, theory-driven evaluation framework [32,35]. They often treat safety, usability, and clinical integration as separate issues, failing to provide a coherent model for assessing whether and how LLMs can genuinely support chronic care from a nursing science perspective [36]. To address this gap, this review introduces Orem’s Self-Care Theory as an analytical lens [37,38]. This theory, which centers on patients’ self-care agency and the nursing systems required to support it, offers a structured way to evaluate LLMs across three critical dimensions: foundational safety and ethics, enablement of self-care, and operational integration into care workflows [36,39]. By applying this theory-driven framework, this review aims to move beyond isolated technical assessments toward a holistic evaluation of LLMs in chronic disease care.

### Objectives

This mixed methods systematic review aims to synthesize quantitative, qualitative, and mixed methods evidence on the application of LLMs in chronic disease care, and to critically evaluate this evidence through a theory-driven 3D framework informed by Orem’s Self-Care Theory (safety and ethics, self-care enablement, and operational and system integration).

## Methods

### Study Design and Registration

This study is a mixed methods systematic review. The review protocol was registered with the International Prospective Register of Systematic Reviews (registration number: CRD420251208327). The reporting of this systematic review followed the PRISMA (Preferred Reporting Items for Systematic Reviews and Meta-Analyses) 2020 statement [40]. The PRISMA 2020 expanded checklist is provided in Checklist 1.

### Information Sources

This review conducted a comprehensive search across 12 electronic databases, including PubMed, Web of Science, Embase, Cochrane Library, CINAHL, Wiley Online Library, SpringerLink, ScienceDirect, China National Knowledge Infrastructure, Wanfang Data, VIP Database, and the China Biology Medicine Disc, from inception to April 2026. Both Chinese and English databases were included to ensure comprehensive coverage of the literature. In addition to the database search, we performed backward and forward citation searching of the reference lists of included studies and relevant reviews to identify additional eligible records.

### Search Strategy

The search strategy was developed collaboratively by the research team (LZ, HL, PH, and JS), all of whom had received standardized training in systematic review search methodology prior to conducting the search. The team first identified key concepts based on the Sample, Phenomenon of Interest, Design, Evaluation, Research type framework and translated these concepts into search terms using Boolean operators (AND, OR, NOT). Medical Subject Headings terms and free-text words were combined to maximize sensitivity and specificity. The preliminary search strategy was then reviewed and validated by an experienced medical librarian to ensure methodological rigor and adherence to best practices in systematic review searching. Following the librarian’s feedback, the final search strategies were refined and finalized by the research team.

The search used Boolean logic with AND to combine the 3 thematic domains (chronic disease, LLMs, and application context), OR to link synonymous terms within each domain, and NOT to exclude irrelevant content when necessary. This structured approach ensured comprehensive retrieval of potentially relevant studies while maintaining acceptable precision. The complete search strategies for all 12 databases, including database-specific syntax adjustments and any applied limits (eg, language restrictions to Chinese and English), are provided in Multimedia Appendix 1 for full transparency and reproducibility.

The reporting of the search strategy followed the PRISMA-S (Preferred Reporting Items for Systematic Reviews and Meta-Analyses literature search extension) guideline [41]. A detailed explanation of how each PRISMA-S item was addressed is provided in Checklist 2.

### Eligibility Criteria

This study used the SPIDER (sample, phenomenon of interest, design, evaluation, research) framework to formulate the inclusion criteria [42]. Eligibility criteria are listed in Textbox 1.

Textbox 1. **Inclusion and exclusion criteria.**

**Inclusion criteria**

- Sample: patients with major chronic noncommunicable diseases as defined by the World Health Organization, including but not limited to cardiovascular diseases (hypertension, coronary heart disease, and stroke), diabetes mellitus (type 1 and type 2), cancers (various types), chronic respiratory diseases (chronic obstructive pulmonary disease and asthma), chronic kidney disease, digestive system diseases (chronic hepatitis, cirrhosis, inflammatory bowel disease, and celiac disease), musculoskeletal disorders (arthritis, fibromyalgia, and axial spondyloarthritis), and neurodegenerative conditions (Parkinson and Alzheimer disease), etc [43]. These disease categories were explicitly incorporated as search keywords to ensure comprehensive coverage of the chronic disease spectrum (see Multimedia Appendix 2).
- Phenomenon of interest: studies where LLMs (eg, general-purpose models like ChatGPT, Gemini, Claude, DeepSeek, Llama, or customized models developed for specific diseases) were applied in activities related to chronic disease care, including but not limited to: clinical decision support, patient health education, self-management support, symptom assessment, risk prediction, data interpretation, and emotional support.
- Design: all types of original research: quantitative (randomized controlled trials, nonrandomized controlled trials, cohort studies, case-control studies, cross-sectional studies, and quasi-experimental studies), qualitative (phenomenology, grounded theory, ethnography, and case studies), and mixed methods studies.
- Evaluation: any outcome measure related to LLM application, including model performance metrics, clinical outcomes, patient-reported outcomes, user experience, and safety indicators.
- Research type: original research published in peer-reviewed journals, with language restricted to Chinese or English.

**Exclusion criteria**

- Studies not involving LLM interventions.
- Studies not addressing chronic disease care.
- Publication types such as reviews, systematic reviews, meta-analyses, conference abstracts, dissertations, commentaries, letters, news reports, and book chapters.
- Articles where full text could not be obtained.
- Studies rated low quality during quality appraisal.

### Selection Process and Data Collection Process

This review used the literature management software EndNote 21 (Clarivate). Two researchers (LZ and HL) independently performed study selection and data extraction. Initial screening was based on titles and abstracts; full-text screening was conducted for potentially relevant studies. For full texts that could not be accessed, attempts were made to contact corresponding authors or obtain copies through institutional library resources. Disagreements were resolved through discussion or by consulting a third researcher (PH).

### Data Items

Data extraction was performed using a predesigned standardized data extraction form, including (1) basic information, (2) study design, (3) disease type or study population, (4) LLM, (5) application scenario, (6) key findings, (7) assessment tools, (8) framework dimension.

### Study Risk-of-Bias Assessment

Methodological quality was assessed using the Mixed Methods Appraisal Tool version 2018 [44]. Based on the ratings, each study was assigned an overall quality rating: “High quality” (meeting all five criteria), “Moderate quality” (meeting four criteria), or “Low quality” (meeting three or fewer criteria). Two researchers (LZ and HL) independently conducted the quality appraisal. First, for each included study, the appropriate Mixed Methods Appraisal Tool category was determined based on its study design. Subsequently, both researchers independently read the full text, evaluated each of the five criteria for the respective category, and completed a predesigned quality assessment form. After completion, the researchers cross-checked their ratings. Disagreements were resolved through discussion. When there was disagreement, the decision was made through joint discussion with the third researcher (PH).

### Synthesis Methods

Due to significant clinical and methodological heterogeneity among the included studies regarding study designs, intervention implementations, and outcome measures, a quantitative meta-analysis was not appropriate [45]. Therefore, this study used thematic synthesis to inductively synthesize the findings from the included literature, reporting in accordance with the Synthesis without meta-analysis guidelines [46]. The Synthesis without meta-analysis Checklist is provided in Checklist 3.

To enhance the analytical depth and theoretical explanatory power of the thematic synthesis, the recently proposed 3D evaluative framework (foundational: safety or privacy or fairness; intermediate: self-care enablement; top: design or system integration), informed by Orem’s Self-Care Theory, was adopted as the theoretical lens. This framework is suitable for systematically analyzing user acceptance, trust, and the clinical integration of LLMs.

The thematic synthesis followed an iterative, 3-step process informed by Thomas and Harden approach [47]. First, line-by-line coding. Two researchers (LZ and PH) independently extracted text segments reporting findings on LLM application, efficacy, or challenges, and assigned descriptive codes using NVivo 14. Intercoder agreement was 92.3% (Cohen κ=0.88). Second, development of descriptive themes. Related codes were grouped (eg, “high accuracy in diagnosis,” “effective for patient education” → “Application efficacy”). Third, generation of analytical themes. Descriptive themes were mapped onto the three dimensions of the Orem-informed framework. For example, codes related to “hallucination risks” and “data security” were interpreted as part of the foundational layer (safety and ethics); codes related to “improved self-efficacy” and “emotional support” were mapped to the intermediate layer (self-care enablement); and codes related to “workflow integration” and “cost-effectiveness” were interpreted as the top layer (design and operational integration). The complete coding table is available in Multimedia Appendix 2.

## Results

### Study Selection

**Figure 1.**
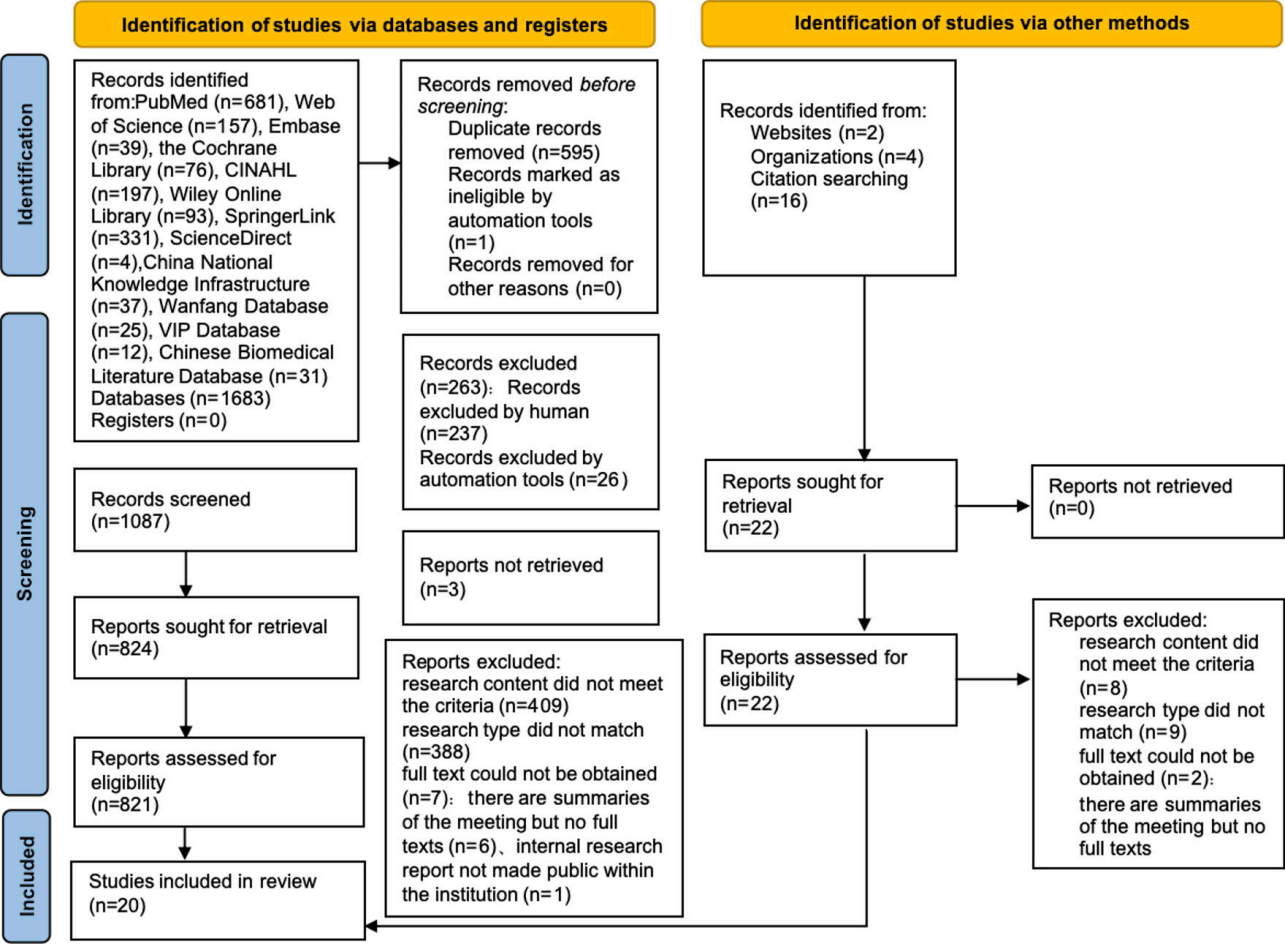
Literature screening process.

The initial search yielded 2164 records (1959 in English and 205 in Chinese). Reports excluded (1) research content did not meet the criteria– that is, the study did not involve patients with chronic noncommunicable diseases as defined in our inclusion criteria, did not apply LLMs as the core intervention, or investigated LLMs for purposes unrelated to chronic disease care (eg, general medical education, administrative tasks, or nonclinical applications); (2) research type did not match–that is, the publication was not an original research article (eg, it was a review, systematic review, meta-analysis, conference abstract, commentary, letter, news report, or book chapter), which did not meet our design inclusion criteria; (3) full text could not be obtained even after attempts to contact corresponding authors and search through institutional library resources. Following the stepwise screening process, 20 studies were ultimately included, all published in English [48-67]. The detailed screening process is illustrated in Figure 1.

### Study Characteristics

**Figure 2.**
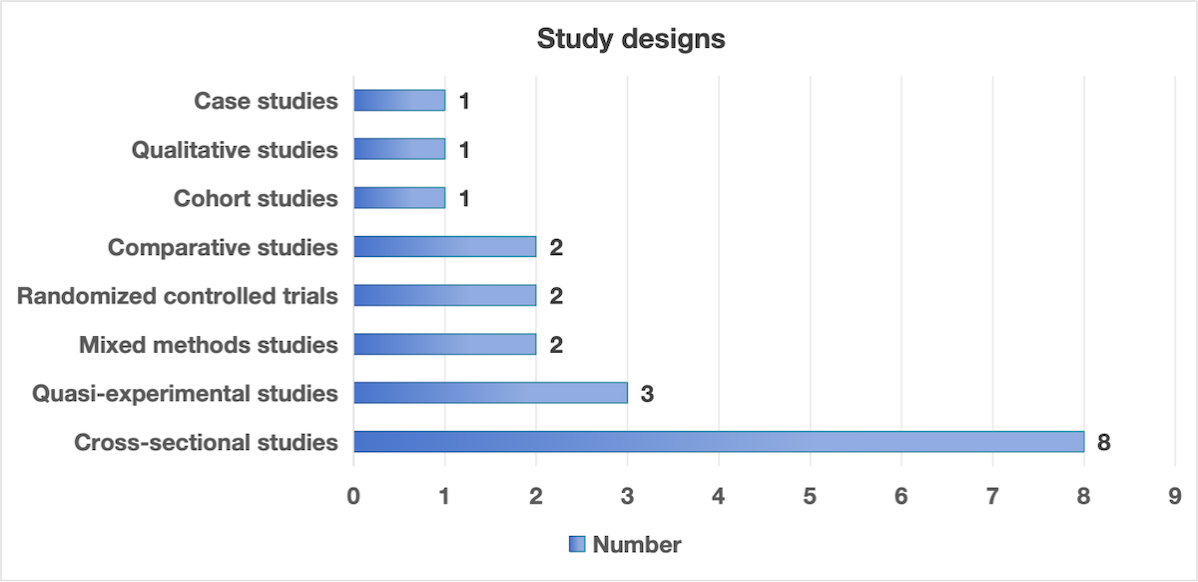
Types of research literature.

**Figure 3.**
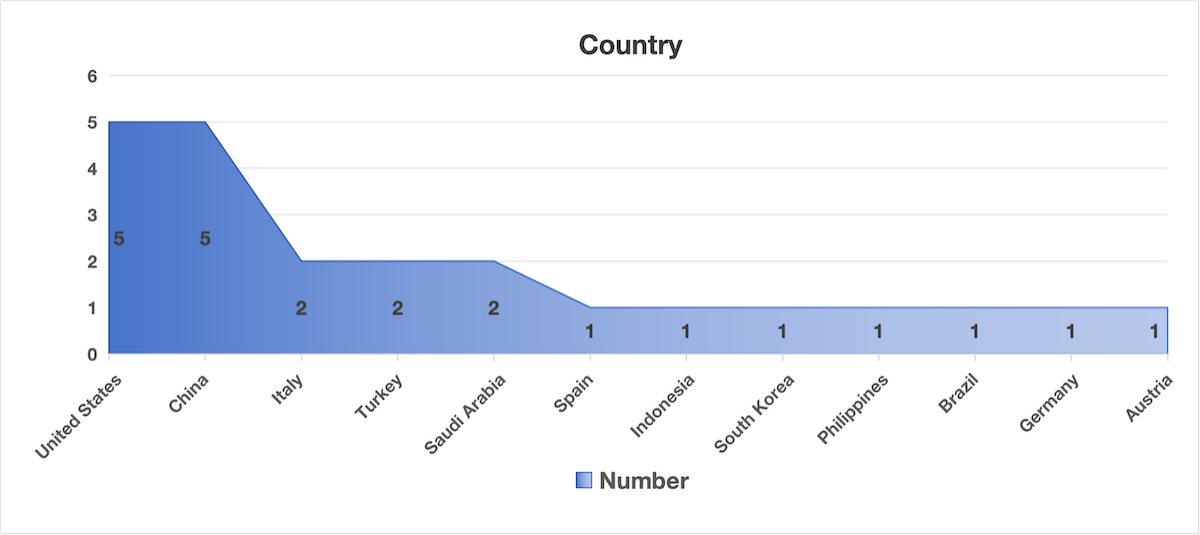
Distribution of the countries included in the literature.

The 20 included studies were published in 2025 (n=17) [48-59,61,63,64,66,67] and 2026 (n=3) [60,62,65], systematically representing recent research progress on LLMs in chronic disease care. A variety of study designs were represented: cross-sectional studies (n=8) [48,50,53,54,57,62,63,67], quasi-experimental studies (n=3) [52,56,58], mixed methods studies (n=2) [50,55], randomized controlled trials (n=2) [60,64], comparative studies (n=2) [65,66], cohort study (n=1) [61], qualitative study (n=1) [58], and case study (n=1) [59]. The number of the types of literature involved in this study is shown in Figure 2. The geographical distribution of the studies was broad, involving the United States (n=5) [51,54,57,59,60], China (n=5) [50,52,53,58,62], Italy (n=2) [51,63], Turkey (n=2) [48,67], Saudi Arabia (n=2) [55,65], Spain (n=1) [49], Indonesia (n=1) [56], South Korea (n=1) [56], the Philippines (n=1) [56], Brazil (n=1) [61], Germany (n=1) [64], and Austria (n=1) [66]. The distribution of the countries included in the literature is shown in Figure 3.

### Results of Individual Studies

**Figure 4.**
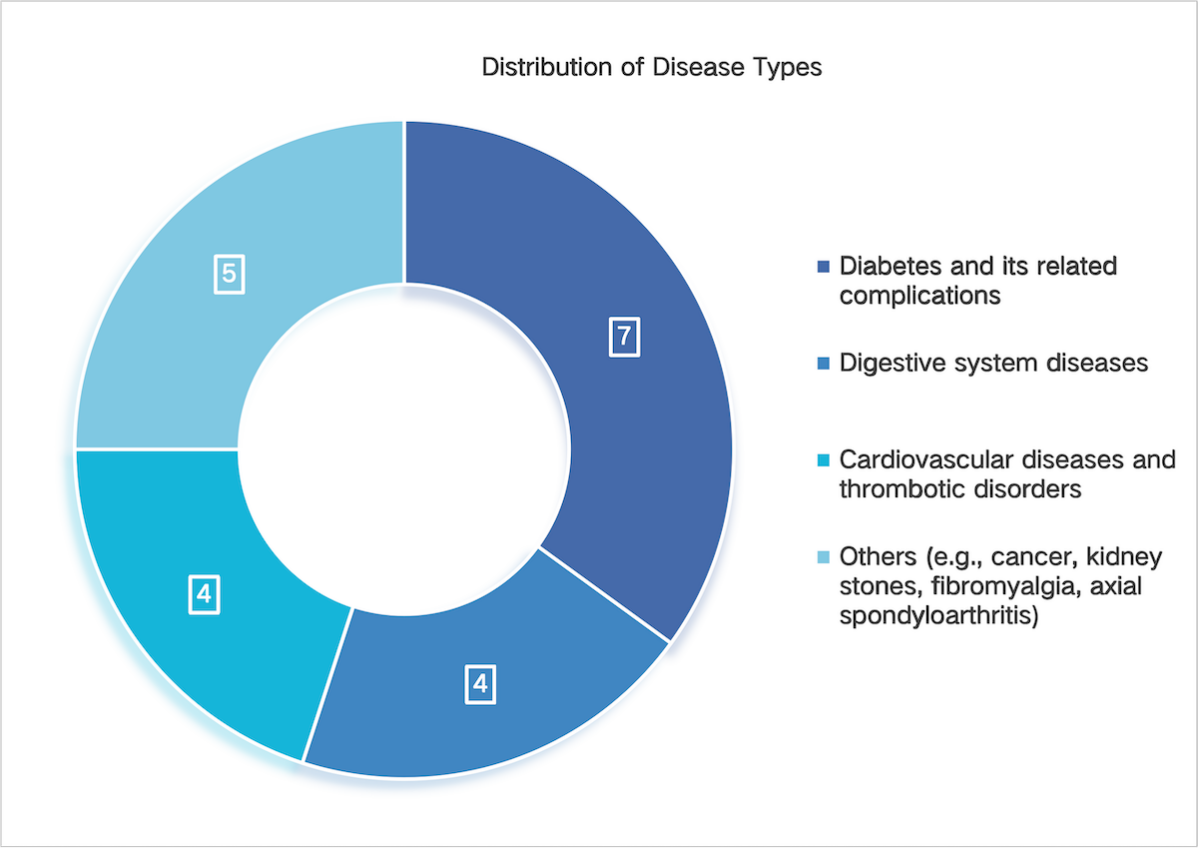
Distribution of disease types.

**Figure 5.**
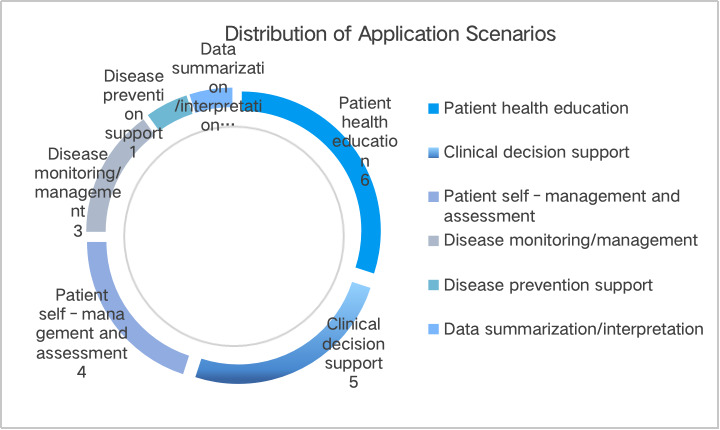
Distribution of application scenarios.

The included studies covered a diverse range of chronic disease types, including diabetes and its related complications (n=7) [55-57,59,61,65,67], digestive system diseases (n=4) [51,52,60,63], cardiovascular diseases and thrombotic disorders (n=4) [54,60,64,66], as well as other conditions such as cancer [58], kidney stones [53], fibromyalgia [49], and axial spondyloarthritis [48]. The distribution of disease types included in this review is shown in Figure 4. Regarding application scenarios, the use of LLMs in chronic disease care primarily focused on patient health education (n=6) [54,55,62,63,66,67], clinical decision support (n=5) [48,51,60,64,66], and patient self-management and assessment (n=4) [49,56,61,65]. Additional studies explored scenarios like data summarization and interpretation [59] and disease prevention support [53]. The distribution of the application scenarios covered by this review is shown in Figure 5. In terms of model selection, most studies used general-purpose LLMs, with OpenAI’s GPT series (including GPT-3.5, GPT-4, GPT-4o, GPT-4 Turbo, GPT-5, etc) being the most widely applied (n=17) [48-53,55,56,58,59,61-67]. Google Gemini series (including Gemini 2.0 Flash, Gemini 2.5 Pro, etc) was also evaluated in several studies (n=8) [48,50,55,57,60,63,66,67]. Furthermore, some studies explored customized or task-specific fine-tuned models, such as GutGPT for gastrointestinal diseases [52], KSrisk-GPT for identifying kidney stone risk factors [53], CARDIO for cardiovascular health education [55], and a Retrieval-Augmented Generation (RAG)-enhanced model for hepatitis C management [51]. Detailed characteristics of the included studies are presented in Table 1.

**Table 1.** Basic characteristics of included literature (n=20).

Author(s), Year	Nation	Study design	Disease type or study population	Large language model	Application scenario	Assessment tools	Key findings	Framework dimension
Usen et al, 2025 [48]	Turkey	Cross-sectional study	Axial spondyloarthritis	ChatGPT-3.5 or 4o, Gemini 2.0 Flash	Clinical decision support	7–point Likert scale, Flesch–Kincaid, ROUGE–L^a^	ChatGPT–4o and Gemini demonstrated superior reliability or usability; improved information accessibility.	Intermediate: clinical decision support+top: readability
Amidei et al, 2025 [49]	Spain	Mixed-methods study	Fibromyalgia, chronic pain	GPT-4	Patient self-assessment	Relevance, RMSE^b^, Gwet AC2, Krippendorff alpha, revised FIQR^c^	High accuracy, comparable to experts; exhibited cross–linguistic adaptability.	Intermediate: self-assessment
Liet al, 2025	China	Cross-sectional study	Hepatitis B virus infection	ChatGPT-3.5 or 4.0, Google Gemini	Patient information support	4–point accuracy scale, Gunning Fog index, Flesch–Kincaid	ChatGPT–4.0 achieved the highest accuracy; however, excessive readability levels limited its usability.	Intermediate: accuracy+top: readability
Giuffrè et al, 2025 [51]	United States, Italy	Quasi-experimental study	Hepatitis C	GPT-4 Turbo	Treatment decision support	10–point Likert scale, Fleiss’ Kappa, ICC^d^, expert consensus	RAG–Top10^e^ achieved 91.7% accuracy, reduced hallucinations, and improved guideline adherence.	Foundational: hallucination mitigation+intermediate: accuracy
Zhang et al, 2025 [52]	China	Quasi-experimental study	Gastrointestinal diseases	GutGPT	Disease diagnosis support	Expert evaluation, ROUGE or BLEU^f^, public datasets	Diagnostic accuracy improved by 9.59%‐22.47%; enhanced patient self–management.	Intermediate: diagnosis support+self-management
Mao et al,2025 [53]	China	Cross-sectional study	Kidney stones	GPT-4.0 (KSrisk-GPT)	Disease prevention support	Accuracy, Precision, Recall, *F*₁-score	KSrisk–GPT identified risk with 95.9% accuracy; improved patient cognition.	Intermediate: risk identification
Rullo et al, 2025 [54]	United States	Mixed-methods study	Cardiovascular disease and HIV infection	CARDIO (fine-tuned Llama 3.1-8B)	Patient health education	BLEU, METEOR^g^, ROUGE, Kincaid, Jargon score, expert evaluation	Fine–tuning improved accuracy, readability, and professionalism; reduced jargon.	Intermediate: accuracy+top: readability
Jamil et al, 2025 [55]	Saudi Arabia	Cross-sectional study	Celiac disease, Type 1 diabetes	ChatGPT 3.5 or 4.0, Google Gemini	Patient health education	3–point accuracy or comprehensiveness scale, Flesch score, Flesch–Kincaid	ChatGPT 4.0 exhibited the best readability and consistency; overall high information accessibility.	Top: readability
Pawana et al, 2025 [56]	Indonesia, South Korea, Philippines	Quasi-experimental study	Diabetes mellitus	LLaMA 3.2, GPT-2, Phi-1, Gemma	Disease management and control	Accuracy, Precision, Recall, *F*_1_-score, Confusion matrix	Fine–tuned LLaMA 3.2 was optimal for anomaly detection; improved monitoring capabilities.	Intermediate: disease monitoring
Kim et al, 2025 [57]	United States	Cross-sectional study	Diabetes mellitus	Llama 3.1, Gemini Pro 1.5, OpenAI o1, DeepSeek R1	Detection of disease symptoms	*F*_1_-score, Precision, Recall, Accuracy, PHQ–^h^4 scale	Most LLMs^i^ achieved >90% accuracy in symptom identification; Llama 3.1 405B performed best.	Intermediate: symptom detection
Zeng et al, 2025 [58]	China	Qualitative study	Cancer	ChatGPT, Kimichat	Patient information inquiry	Semi–structured interviews, Colaizzi’s method of analysis	Physicians acknowledged management potential but concerns regarding liability and misinformation persisted.	Foundational: ethical liability+intermediate: potential
Healey et al, 2025 [59]	United States	Case study	Type 1 diabetes (CGM analysis)	GPT-4 (Data Analyst)	CGM data summarization	Accuracy or Completeness or Safety or Appropriateness ratings, Gwet’s AC1	Perfect scores on 9/10 quantitative metrics; high scores (8–10/10) for accuracy, completeness, and safety in qualitative summaries.	Foundational: safety+Intermediate: data summarization
O'Sullivan et al, 2026 [60]	United States	Randomized controlled study	Inherited cardiomyopathies	AMIE (Gemini 2.0 Flash)	Clinical decision support	10–domain preference assessment, error analysis, self–reported time savings	Physicians assisted by AMIE were preferred by experts, made fewer errors (24.3% vs 13.1%), and had fewer omissions (37.4% vs 17.8%).	Intermediate: clinical decision support
Furtado et al, 2025 [61]	Brazil	Cohort study	Type 2 diabetes	GPT-3.5-based MarIA	Patient management support	Engagement metrics, expert qualitative assessment, user questionnaires	Personalization increased engagement by 26% and quadrupled message length; no hallucinations but general advice required caution.	Foundational: no hallucinations +intermediate: personalization
Zhang et al, 2026 [62]	China	Cross-sectional study	Skin cancer	Doubao, DeepSeek, Wenxin Yiyan, Tongyi Qianwen, GPT-5	Patient health education	c–PEMAT–P^j^, GQS^k^, 7 readability metrics	GPT–5 had the highest GQS score; readability varied significantly among models, showing weak correlation with quality.	Top: readability
Bertani et al, 2025 [63]	Italy	Cross-sectional study	Celiac disease	ChatGPT-4, Claude 3.7, Gemini 2.0	Patient health education	5–point accuracy or clarity scale, readability metrics, misinformation detection	Gemini was optimal for accuracy, clarity, and readability; however, all models exhibited 13%‐24% misinformation.	Foundational: misinformation
Carl et al, 2025 [64]	Germany	Randomized controlled study	Urological tumors	UroBot (GPT-4o+RAG) vs. ChatGPT	Clinical decision support	4–domain assessment, preference Likert scale, Krippendorff alpha	UroBot was superior in correctness of recommendations (73% vs 50%), source attribution (74% vs 30%), and verifiability (84% vs 35%); physicians trusted it more.	Foundational: source attribution+intermediate: trust
Alredaini et al, 2026 [65]	Saudi Arabia	Comparative study	Type 2 diabetes (blood glucose prediction)	GPT-4.1, MiniGPT, LLaMA-1B, LLaMA-7B, traditional or deep learning models	Patient disease prediction	MAE^l^, RMSE, MAPE^m^, R²^n^, SHAP^o^, GPT explanation	GPT–4.1 performed best at 30/60 minutes; LLaMA–7B at 90 minutes; LLMs outperformed other models.	Intermediate: glucose prediction
Vladic et al, 2025 [66]	Austria	Comparative study	Venous thromboembolism	Le Chat Pixtral Large, DeepSeek-R1, ChatGPT-4.5	Patient health education, Clinical decision support	10–point adequacy scale, identifiability scale, potential harm assessment	LLMs provided superior patient education compared to experts; DeepSeek–R1 outperformed experts in clinical decision–making; physicians could not distinguish.	Intermediate: patient education+clinical decision
Yigit Yalcin et al, 2025 [67]	Turkey	Cross-sectional study	Gestational diabetes	ChatGPT-4o, DeepSeek R-1, Gemini 2.5 Pro, Grok 3.0	Patient health education	mDISCERN^p^, GQS, readability metrics, TTR^q^	Grok and Gemini scored highest on mDISCERN or GQS; DeepSeek had the best readability, but all FRES scores were <60.	Top: readability

a ROUGE–L: Recall-Oriented Understudy for Gisting Evaluation – Longest Common Subsequence.

b RMSE: root mean square error.

c FIQR: Revised Fibromyalgia Impact Questionnaire.

d ICC: Intraclass Correlation Coefficient.

e RAG: retrieval-augmented generation.

f BLEU: Bilingual Evaluation Understudy.

g METEOR: Metric for Evaluation of Translation with Explicit ORdering.

h PHQ: Patient Health Questionnaire.

i LLM: large language model.

j c–PEMAT–P: Chinese Version of the Patient Education Materials Assessment Tool for Printable Materials

k GQS: Global Quality Score.

l MAE: mean absolute error.

m MAPE: mean absolute percentage error.

n R²: coefficient of determination.

o SHAP: Shapley Additive Explanations.

p mDISCERN: Modified DISCERN.

q TTR: type-token ratio.

### Reporting Biases

All 20 included studies met the inclusion criteria and underwent rigorous quality assessment. All were rated as moderate or high quality; no studies were rated as low quality. The detailed results of the methodological quality appraisal are shown in Table 2.

**Table 2.** Methodological quality assessment of included studies using the mixed methods appraisal.

Author(s), Year	Study design (MMAT^a^ category)	S1^b^	S2	Q1^c^	Q2	Q3	Q4	Q5	Overall quality
Usen et al, 2025 [48]	Quantitative descriptive (4)	Yes	Yes	Yes	Yes	Yes	Yes	Yes	High
Amidei et al, 2025 [49]	Mixed methods (5)	Yes	Yes	Yes	Yes	Yes	Yes	Unclear	Moderate
Li et al, 2025 [50]	Quantitative descriptive (4)	Yes	Yes	Yes	Yes	Yes	Yes	Yes	High
Giuffrè et al, 2025 [51]	Quantitative nonrandomized (3)	Yes	Yes	Unclear	Yes	Yes	Yes	Yes	Moderate
Zhang et al, 2025 [52]	Quantitative nonrandomized (3)	Yes	Yes	Yes	Yes	Yes	Yes	Yes	High
Mao et al,2025 [53]	Quantitative descriptive (4)	Yes	Yes	Yes	Yes	Yes	Yes	Yes	High
Rullo et al, 2025 [54]	Mixed methods (5)	Yes	Yes	Yes	Yes	Yes	Yes	Yes	High
Jamil et al, 2025 [55]	Quantitative descriptive (4)	Yes	Yes	Yes	Yes	Yes	Yes	Yes	High
Pawana et al, 2025 [56]	Quantitative nonrandomized (3)	Yes	Yes	Unclear	Yes	Yes	Yes	Yes	Moderate
Kim et al, 2025 [57]	Quantitative descriptive (4)	Yes	Yes	Yes	Yes	Yes	Yes	Yes	High
Zeng et al, 2025 [58]	Qualitative research (1)	Yes	Yes	Yes	Yes	Yes	Yes	Yes	High
Healey et al, 2025 [59]	Mixed methods (5)	Yes	Yes	Yes	Yes	Yes	Yes	Yes	High
O'Sullivan et al, 2026 [60]	Randomized controlled trial (2)	Yes	Yes	Yes	Yes	Yes	Yes	Yes	High
Furtado et al, 2025 [61]	Quantitative nonrandomized (3)	Yes	Yes	Yes	Yes	Yes	No	Yes	Moderate
Zhang et al, 2026 [62]	Quantitative descriptive (4)	Yes	Yes	Yes	Yes	Yes	Yes	Yes	High
Bertani et al, 2025 [63]	Quantitative descriptive (4)	Yes	Yes	Yes	Yes	Yes	Yes	Yes	High
Carl et al, 2025 [64]	Randomized controlled trial (2)	Yes	Yes	Yes	Yes	Yes	Yes	Yes	High
Alredaini et al, 2026 [65]	Quantitative nonrandomized (3)	Yes	Yes	Yes	Yes	Yes	Yes	Yes	High
Vladic et al, 2025 [66]	Quantitative nonrandomized (3)	Yes	Yes	Unclear	Yes	Yes	Yes	Yes	Moderate
Yigit Yalcin et al, 2025 [67]	Quantitative descriptive (4)	Yes	Yes	Yes	Yes	Yes	Yes	Yes	High

a MMAT: Mixed Methods Appraisal Tool.

b S: screening questions.

c Q: quality criteria.

### Results of Syntheses

The thematic synthesis, informed by the Orem-based 3D evaluative framework, generated three analytical themes corresponding to the framework’s core layers.

1. Theme 1: foundational layer: safety, privacy, and fairness as prerequisites. This theme captures the nonnegotiable baseline requirements for LLM deployment. Across studies, primary challenges included hallucination risks (ie, generation of factually incorrect information), data privacy and security concerns, and ambiguous ethical responsibility for AI-generated recommendations. For instance, Bertani et al [63] found that all tested models exhibited 13%‐24% misinformation when providing patient education for celiac disease. From the perspective of the 3D framework, these issues represent failures in the foundational layer. Without robust safeguards against hallucinations and breaches of privacy, LLMs cannot be considered safe for integration into chronic care, regardless of their performance in other areas [68].
2. Theme 2: intermediate layer: self-care agency enablement through perceived usefulness and trust. This theme reflects the LLM’s ability to meet self-care needs and enhance patient capabilities. Patient health education (n=6) emerged as the most common application [54,55,62,63,66,67]. For instance, Jamil et al [55] found ChatGPT-4.0 superior in readability and consistency for celiac disease and type 1 diabetes education. Clinical decision support (n=5) represented another core domain [48,51,60,64,66]. O’Sullivan et al.’s [60] randomized controlled trial showed that physicians assisted by AMIE (Google Research) made fewer errors. Patient self-management and assessment (n=4) was an emerging domain [49,56,61,65]. Amidei et al [49] found GPT-4 comparable to experts in pain self-assessment for fibromyalgia. From the framework’s perspective, these scenarios demonstrate the LLM’s perceived usefulness in providing cognitive support, thereby contributing to the patient’s self-care agency [39]. Three key drivers of enhanced efficacy were identified in this layer: (a) foundation model iteration (eg, GPT-4 series outperformed GPT-3.5); (b) RAG, which improved accuracy from 36.6% to 91.7% in one hepatitis C study; (c) specialized fine-tuning, which improved diagnostic accuracy by 9.6%‐−22.5% for GutGPT [51,61,64]. Within the framework, RAG and finding primarily enhance perceived usefulness and trust, thereby solidifying the LLM’s role in enabling self-care.
3. Theme 3: top layer: design adaptability and system integration. This final theme concerns LLM’s usability and its seamless integration into real-world care workflows. Multiple studies reported that the reading difficulty of LLM-generated content often exceeded patient comprehension levels (eg, Flesch-Kincaid grade levels >10) [48,50,54]. This readability mismatch represents a barrier to perceived ease of use, a key component of design adaptability, particularly for patients with lower health literacy [30]. Furthermore, some studies evaluated LLMs in controlled, siloed settings, with little evidence on their integration into existing nursing workflows, impact on nursing workload, or long-term cost-effectiveness [48-55,67]. These gaps highlight the underdeveloped nature of the top layer of the evaluation framework, limiting clinical adoption [69].

### Evidence Distribution Across the 3D Framework

**Figure 6.**
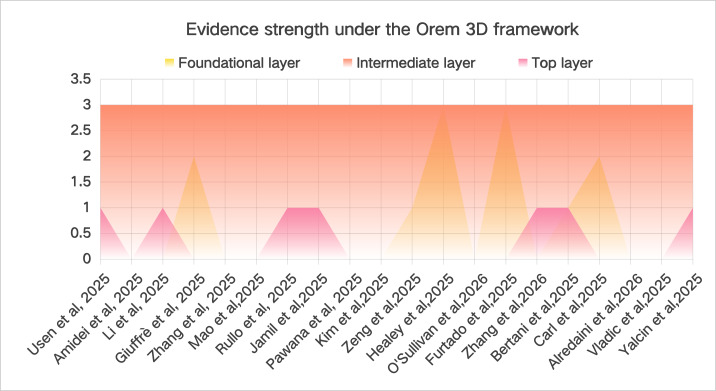
The area chart of evidence strength under the Orem 3D framework [48-67].

To visually demonstrate the coverage, intensity, and gap situation of the evidence based on Orem’s 3D framework, this review has constructed an area chart (Figures 4 and 6). The x-axis represents individual studies; the y-axis represents the base layer (safety or privacy or equity: illusion risk, data privacy, ethical responsibility, algorithm fairness, etc), the middle layer (self-care ability: patient education, clinical decision support, self-management or monitoring, etc), and the top layer (design and system integration: readability matching, workflow integration, cost-effectiveness, etc).

In the foundational layer, hallucination risk and ethical responsibility show moderate evidence, but data privacy and algorithmic fairness are largely unassessed. The intermediate layer has the strongest evidence, especially in patient education, clinical decision support, and self-management, confirming that LLMs primarily enable self-care under controlled conditions. The top layer shows critical gaps: readability matching is weak, while workflow integration and cost-effectiveness are almost entirely absent. Thus, the evidence is unbalanced—strong in the intermediate layer, weak in foundational and top layers. Future research should prioritize foundational safety or fairness validation and top-layer integration and economic evaluations.

## Discussion

### Summary of Main Findings

This mixed methods systematic review synthesized 20 studies on LLMs in chronic disease care and evaluated them through a theory-driven, 3D framework informed by Orem’s Self-Care Theory [48-54,59,61-64,70]. Three main findings emerged. First, foundational safety, privacy, and fairness issues remain persistent barriers. Hallucinations, data security concerns, and potential algorithmic biases were consistently reported across studies, indicating that current LLMs do not yet meet the baseline requirements for safe deployment in chronic care. Second, LLMs demonstrate clear potential to enable patient self-care agency within the intermediate layer. Evidence was strongest for patient health education, clinical decision support, and self-management assistance, with technical enhancements (RAG, fine-tuning, and model iteration) further improving perceived usefulness and trust. Third, the top layer of the framework—design adaptability and system integration—is critically underdeveloped. Readability mismatches between LLM-generated content and patient health literacy levels were ubiquitous, yet evidence on workflow integration, nursing workload, cost-effectiveness, and long-term sustainability was almost entirely absent.

In summary, current LLM applications in chronic disease care have proven their value primarily within controlled technical validation settings (intermediate layer), but lack foundational safety assurances and operational integration. This review provides a structured, theory-informed roadmap for future research to address these critical gaps.

### Comparison With Related Studies

The findings of this review both complement and extend prior systematic evidence. Previous work has established the feasibility of LLMs in chronic disease management, yet several important limitations remain. Li et al [33] conducted a mixed methods review focusing primarily on quantitative feasibility metrics. While their work provided a valuable overview, it did not integrate established nursing theories to ground the analysis in core principles of self-care. Consequently, their evaluation remained at the level of technical performance without a structured, multidimensional framework. Watson et al [34] performed an integrative review of generative AI in general nursing practice. Although comprehensive in scope, their review did not specifically address the unique demands of chronic disease care, nor did it offer a theory-driven approach to assess how LLMs might support patient self-care agencies or integrate into existing nursing workflows.

This review addresses these gaps directly. By adopting Orem’s Self-Care Theory as an analytical lens and operationalizing it through a 3D evaluative framework—foundational safety and ethics, self-care enablement, and operational integration—move beyond isolated performance metrics to provide a structured, clinically meaningful assessment of LLM readiness for chronic care. Moreover, this review focuses on high-burden chronic conditions, and the inclusion of both quantitative and qualitative evidence allows for a more granular, human-centric critique of current applications, revealing not only where LLMs show promise but also where critical gaps persist.

### Evidence Distribution and Study Characteristics

This systematic review of the mixed-method systematically collated and evaluated 20 studies, revealing the current application status, core efficacy, and challenges of LLMs in chronic disease care [48-54,59,61-64,70].

According to World Health Organization statistics, chronic diseases are the leading cause of death globally, accounting for approximately 41 million deaths annually, or 74% of all deaths [71]. Among these, diabetes, cardiovascular diseases, cancer, and chronic respiratory diseases constitute the bulk of this burden, affecting the quality of life and health expectancy of billions worldwide [72]. Given that chronic disease care is characterized by frequent daily monitoring, high dependence on behavioral interventions, and continuous health information needs, accessibility and powerful information integration and personalized generation capabilities of LLMs offer potential solutions to these persistent, dynamic care challenges [73]. In terms of disease spectrum, LLM applications have covered various chronic conditions, including diabetes, cardiovascular disease, digestive system diseases, cancer, and chronic pain, demonstrating their adaptability across diverse disease contexts [50-52,55,57-59,61-63,66,67]. Diabetes and its related complications were the most studied area (n=7) [55-57,59,61,65,67], which is consistent with its status as one of the world’s major chronic diseases with complex management demands reliant on continuous monitoring and behavioral intervention [27,74]. However, despite cardiovascular and chronic respiratory diseases being major sources of global disease burden, the number of relevant studies was relatively low, suggesting a need for increased focus on these high-burden conditions in future research [75,76].

Regarding study design, the included literature predominantly consisted of quantitative descriptive studies (n=8) [48,50,53,55,57,62,63,67] and nonrandomized quantitative studies (n=5) [52,56,58,65,66], with fewer randomized controlled trials [60,64], mixed methods [49,55], and qualitative studies [58]. This distribution reflects that the current field is still in a preliminary stage, primarily focused on technical validation and efficacy exploration [77]. Qualitative and mixed methods of research hold unique value in understanding user (both patient and health care provider) acceptance, usage experiences, and potential socio-cultural barriers to new technologies [78]. This is particularly crucial during the early integration of novel technologies like LLMs into complex clinical environments, where in-depth exploration of nurse-patient interaction, trust-building, and ethical dilemmas from a “human-centric” perspective is essential to guide technical optimization and practical translation [79-81]. Future studies should incorporate more such designs to complement quantitative insights with deeper contextual understanding.

Geographically, the included studies originated mainly from countries such as Turkey, the United States, Italy, China, and Germany, indicating a shared global interest in LLM applications [48,50-53,55,57-60,62,63,67]. However, this also highlights that current evidence predominantly comes from high-income countries or regions, with underrepresentation from low- and middle-income countries or medically underserved areas [82,83]. Given that factors like health literacy levels, health care resource accessibility, and cultural beliefs can significantly influence the effectiveness of LLM applications, future research should conduct localized studies across diverse health care settings to promote global health equity in technology deployment [84,85].

### Drivers of Intermediate-Layer Performance

This study identified three drivers of improved technical performance: foundation model iteration, RAG, and specialized fine-tuning. The research indicates RAG enhances clinicians’ perceived usefulness by improving accuracy and source verifiability [86-88]. Fine-tuning enhances users’ perceived usefulness by tailoring outputs to specific specialty knowledge needs [89]. However, the finding by Li et al [50] that ChatGPT-4.0 had the highest accuracy but excessive readability levels illustrates that high technical performance within the intermediate layer does not guarantee success in the “top layer” (design adaptability). Thus, future technical development should simultaneously address both cognitive accuracy (intermediate layer) and user-centric design (top layer) [90-92].

### Documented Challenges and the Foundational Layer: Safety, Privacy, Fairness

Primary challenges consistently reported across studies align with the “foundational layer” of framework [50,61,66]. Viewed through the lens of Orem’s theory, these are not merely technical glitches but fundamental threats to the nursing system’s ability to provide safe and ethical care [93]. Hallucinations, for instance, could actively undermine a patient’s self-care agency by providing dangerous misinformation [94]. Data privacy breaches violate the core ethical principle of nonmaleficence [95-97]. These foundational issues should be resolved before LLMs can reliably support any form of compensated or supportive nursing system [98-100].

### Future Research Directions

Based on the evidence gaps identified in this review, future research should prioritize the neglected “top layer” of framework: (1) clinical effectiveness trials measuring patient-centered outcomes (eg, disease control, quality of life) to assess the real-world value of LLMs beyond technical performance [101] [102]; (2) implementation science studies that evaluate the integration of LLMs into existing nursing workflows, including their impact on nursing workload, cost-effectiveness, and scalability [103,104]; (3) readability-adaptive interfaces that can tailor output to individual patient health literacy levels, directly addressing the “design adaptability” sub-dimension [105,106]; (4) safety assurance systems incorporating real-time hallucination detection and verification mechanisms to secure the “foundational layer” [107]; (5) standardized evaluation frameworks developed through interdisciplinary consensus to capture all three dimensions of LLM performance.

### Limitations

Although this mixed methods systematic review strictly adhered to evidence-based research methodologies, several limitations should be acknowledged: (1) the search was limited to published Chinese and English language literature, excluding gray literature, commentaries, books, and news reports, which may introduce publication bias. In addition, although attempts have been made to contact the authors, there are still some articles whose full texts could not be fully retrieved during the search process; (2) despite strict adherence to methodological protocols, the possibility of subjective bias during the synthesis process cannot be entirely ruled out; (3) the study populations originated from various countries with differences in economic status, national policies, and cultural backgrounds, which may affect the comprehensiveness and cross-cultural applicability of the conclusions.

### Conclusion

Applying a novel, Orem-informed 3D framework, this mixed methods systematic review concludes that the current evidence on LLMs in chronic disease care primarily supports the intermediate layer—that is, self-care enablement through useful and trustworthy tools. Within this layer, LLMs demonstrate promising technical performance in areas such as patient education, clinical decision support, and self-management assistance under controlled conditions. However, persistent challenges related to the foundational layer (safety, privacy, and fairness) remain, and evidence for the top layer (design adaptability and system integration) is still limited. These gaps may constrain the readiness of LLMs for routine clinical implementation at present. Future research would benefit from prioritizing clinical effectiveness trials, implementation science, and patient-centered design to address these shortcomings.

## Supplementary material

10.2196/90744 Multimedia Appendix 1 The complete search formula for all databases.

10.2196/90744 Multimedia Appendix 2 Subject comprehensive coding table.

10.2196/90744 Checklist 1 PRISMA 2020 expanded checklist.

10.2196/90744 Checklist 2 PRISMA-S checklist.

10.2196/90744 Checklist 3 SWiM checklist.

## Abbreviations

LLM: large language model
PRISMA: Preferred Reporting Items for Systematic reviews
PRISMA-S: Preferred Reporting Items for Systematic reviews and Meta-Analyses literature search extension
RAG: retrieval-augmented generation
SPIDER: sample, phenomenon of interest, design, evaluation, research
SWiM: Synthesis Without Meta-Analysis
