# Supplementary material for: Application of Large Language Models in Chronic Disease Care: Mixed Methods Systematic Review and Thematic Synthesis
Source: J Med Internet Res. 2026 Aug 11;28:e90744. doi: 10.2196/90744 (PMC13460802; doi:10.2196/90744)
Supplement: Supplementary file 1 [file jmir-v28-e90744-s001.docx]

**PubMed（n=845）**

| Step | Search expression |
| --- | --- |
| #1 | "Large Language Model"[MeSH Major Topic] |
| #2 | "large language model"[Title/Abstract] OR "LLM"[Title/Abstract] OR "LLMs"[Title/Abstract] OR "ChatGPT"[Title/Abstract] OR "GPT-4"[Title/Abstract] OR "Gemini"[Title/Abstract] OR "Claude"[Title/Abstract] OR "DeepSeek"[Title/Abstract] OR "Llama"[Title/Abstract] OR "generative AI"[Title/Abstract] |
| #3 | #1 OR #2 |
| #4 | "Chronic Disease"[MeSH] |
| #5 | "chronic disease"[Title/Abstract] OR "chronic diseases"[Title/Abstract] OR "chronic illness"[Title/Abstract] OR "chronic condition"[Title/Abstract] OR "non-communicable disease"[Title/Abstract] OR "NCD"[Title/Abstract] |
| #6 | "Diabetes Mellitus"[MeSH] OR "Hypertension"[MeSH] OR "Cardiovascular Diseases"[MeSH] OR "Neoplasms"[MeSH] OR "Pulmonary Disease, Chronic Obstructive"[MeSH] OR "Renal Insufficiency, Chronic"[MeSH] OR "Liver Diseases"[MeSH] OR "Digestive System Diseases"[MeSH] OR "Musculoskeletal Diseases"[MeSH] OR "Nervous System Diseases"[MeSH] |
| #7 | "diabetes"[Title/Abstract] OR "hypertension"[Title/Abstract] OR "cardiovascular disease"[Title/Abstract] OR "cancer"[Title/Abstract] OR "tumor"[Title/Abstract] OR "COPD"[Title/Abstract] OR "chronic kidney disease"[Title/Abstract] OR "CKD"[Title/Abstract] OR "liver disease"[Title/Abstract] OR "cirrhosis"[Title/Abstract] OR "hepatitis"[Title/Abstract] OR "digestive disease"[Title/Abstract] OR "IBD"[Title/Abstract] OR "Crohn"[Title/Abstract] OR "arthritis"[Title/Abstract] OR "fibromyalgia"[Title/Abstract] OR "chronic pain"[Title/Abstract] OR "neurodegenerative"[Title/Abstract] OR "Parkinson"[Title/Abstract] OR "Alzheimer"[Title/Abstract] |
| #8 | #4 OR #5 OR #6 OR #7 |
| #9 | "Disease Management"[MeSH] OR "Patient Care"[MeSH] OR "Self Care"[MeSH] OR "Patient Education as Topic"[MeSH] |
| #10 | "care"[Title/Abstract] OR "management"[Title/Abstract] OR "nursing"[Title/Abstract] OR "patient education"[Title/Abstract] OR "self-management"[Title/Abstract] OR "decision support"[Title/Abstract] |
| #11 | #9 OR #10 |
| #12 | #3 AND #8 AND #11 |

**Web of Science（n=233)**

| Step | Search expression |
| --- | --- |
| #1 | TS=("large language model" OR "LLM" OR "LLMs" OR "ChatGPT" OR "GPT-4" OR "GPT-4o" OR "Gemini" OR "Claude" OR "DeepSeek" OR "Llama" OR "generative AI") |
| #2 | TS=("chronic disease" OR "chronic diseases" OR "chronic illness" OR "chronic condition" OR "non-communicable disease" OR "NCD" OR "diabetes" OR "hypertension" OR "cardiovascular disease" OR "heart disease" OR "stroke" OR "cancer" OR "tumor" OR "neoplasm" OR "COPD" OR "chronic respiratory disease" OR "asthma" OR "chronic kidney disease" OR "CKD" OR "renal insufficiency" OR "liver disease" OR "cirrhosis" OR "hepatitis" OR "digestive disease" OR "IBD" OR "Crohn" OR "celiac" OR "arthritis" OR "fibromyalgia" OR "axial spondyloarthritis" OR "musculoskeletal" OR  "Parkinson" OR "Alzheimer" OR "neurodegenerative") |
| #3 | TS=("care" OR "management" OR "nursing" OR "patient education" OR "self-management" OR "decision support" OR "health education") |
| #4 | #1 AND #2 AND #3 |

**Embase（n=64）**

('large language model':ab,ti OR llm:ab,ti OR llms:ab,ti OR chatgpt:ab,ti OR 'gpt 4':ab,ti OR 'gpt 4o':ab,ti OR gemini:ab,ti OR claude:ab,ti OR deepseek:ab,ti OR llama:ab,ti OR 'generative ai':ab,ti) AND ('chronic disease':ab,ti OR 'chronic diseases':ab,ti OR 'chronic illness':ab,ti OR 'chronic condition':ab,ti OR 'non-communicable disease':ab,ti OR ncd:ab,ti OR diabetes:ab,ti OR hypertension:ab,ti OR 'cardiovascular disease':ab,ti OR 'heart disease':ab,ti OR stroke:ab,ti OR cancer:ab,ti OR tumor:ab,ti OR neoplasm:ab,ti OR copd:ab,ti OR 'chronic respiratory disease':ab,ti OR asthma:ab,ti OR 'chronic kidney disease':ab,ti OR ckd:ab,ti OR 'renal insufficiency':ab,ti OR 'liver disease':ab,ti OR cirrhosis:ab,ti OR hepatitis:ab,ti OR 'digestive disease':ab,ti OR ibd:ab,ti OR crohn:ab,ti OR celiac:ab,ti OR arthritis:ab,ti OR fibromyalgia:ab,ti OR 'axial spondyloarthritis':ab,ti OR musculoskeletal:ab,ti OR parkinson:ab,ti OR alzheimer:ab,ti OR neurodegenerative:ab,ti) AND (care:ab,ti OR management:ab,ti OR nursing:ab,ti OR 'patient education':ab,ti OR 'self management':ab,ti OR 'decision support':ab,ti OR 'health education':ab,ti)

**Cochrane Library（n=93）**

("large language model" OR "LLM" OR "LLMs" OR "ChatGPT" OR "GPT-4" OR "GPT-4o" OR "Gemini" OR "Claude" OR "DeepSeek" OR "Llama" OR "generative AI"):ti,ab,kw AND ("chronic disease" OR "chronic diseases" OR "chronic illness" OR "chronic condition" OR "non-communicable disease" OR "NCD" OR "diabetes" OR "hypertension" OR "cardiovascular disease" OR "heart disease" OR "stroke" OR "cancer" OR "tumor" OR "neoplasm" OR "COPD" OR "chronic respiratory disease" OR "asthma" OR "chronic kidney disease" OR "CKD" OR "renal insufficiency" OR "liver disease" OR "cirrhosis" OR "hepatitis" OR "digestive disease" OR "IBD" OR "Crohn" OR "celiac" OR "arthritis" OR "fibromyalgia" OR "axial spondyloarthritis" OR "musculoskeletal" OR "Parkinson" OR "Alzheimer" OR "neurodegenerative"):ti,ab,kw AND

("care" OR "management" OR "nursing" OR "patient education" OR "self-management" OR "decision support" OR "health education"):ti,ab,kw

**CINAHL（n=218）**

XB ("large language model" OR "LLM" OR "LLMs" OR "ChatGPT" OR "GPT-4" OR "GPT-4o" OR "Gemini" OR "Claude" OR "DeepSeek" OR "Llama" OR "generative AI") AND XB ("chronic disease" OR "chronic diseases" OR "chronic illness" OR "chronic condition" OR "non-communicable disease" OR "NCD" OR "diabetes" OR "hypertension" OR "cardiovascular disease" OR "heart disease" OR "stroke" OR "cancer" OR "tumor" OR "neoplasm" OR "COPD" OR "chronic respiratory disease" OR "asthma" OR "chronic kidney disease" OR "CKD" OR "renal insufficiency" OR "liver disease" OR "cirrhosis" OR "hepatitis" OR "digestive disease" OR "IBD" OR "Crohn" OR "celiac" OR "arthritis" OR "fibromyalgia" OR "axial spondyloarthritis" OR "musculoskeletal" OR "Parkinson" OR "Alzheimer" OR "neurodegenerative") AND ("care" OR "management" OR "nursing" OR "patient education" OR "self-management" OR "decision support" OR "health education")

**Wiley Online Library（n=99）**

("large language model" OR "LLM" OR "LLMs" OR "ChatGPT" OR "GPT-4" OR "GPT-4o" OR "Gemini" OR "Claude" OR "DeepSeek" OR "Llama" OR "generative AI") AND ("chronic disease" OR "chronic diseases" OR "chronic illness" OR "chronic condition" OR "non-communicable disease" OR "NCD" OR "diabetes" OR "hypertension" OR "cardiovascular disease" OR "heart disease" OR "stroke" OR "cancer" OR "tumor" OR "neoplasm" OR "COPD" OR "chronic respiratory disease" OR "asthma" OR "chronic kidney disease" OR "CKD" OR "renal insufficiency" OR "liver disease" OR "cirrhosis" OR "hepatitis" OR "digestive disease" OR "IBD" OR "Crohn" OR "celiac" OR "arthritis" OR "fibromyalgia" OR "axial spondyloarthritis" OR "musculoskeletal" OR "Parkinson" OR "Alzheimer" OR "neurodegenerative") AND ("care" OR "management" OR "nursing" OR "patient education" OR "self-management" OR "decision support" OR "health education")

**SpringerLink（n=396）**

Title: ("large language model" OR "LLM" OR "LLMs") AND Keywords: ("chronic disease" OR "chronic diseases" OR "chronic illness" OR "chronic condition" OR "non-communicable disease" OR "NCD" OR "diabetes" OR "hypertension" OR "cardiovascular disease" OR "heart disease" OR "stroke" OR "cancer" OR "tumor" OR "neoplasm" OR "COPD" OR "chronic respiratory disease" OR "asthma" OR "chronic kidney disease" OR "CKD" OR "renal insufficiency" OR "liver disease" OR "cirrhosis" OR "hepatitis" OR "digestive disease" OR "IBD" OR "Crohn" OR "celiac" OR "arthritis" OR "fibromyalgia" OR "axial spondyloarthritis" OR "musculoskeletal" OR "Parkinson" OR "Alzheimer" OR "neurodegenerative") AND (care OR management OR nursing OR "patient education" OR "self-management" OR "decision support" OR "health education")

ScienceDirect（n=11）

("large language model" OR "LLM" OR "LLMs") AND ("chronic disease" OR "chronic diseases" OR "chronic illness" OR "chronic condition" OR "non-communicable disease" OR "NCD" OR "diabetes" OR "hypertension" OR "cardiovascular disease" OR "heart disease" OR "stroke" OR "cancer" OR "tumor" OR "neoplasm" OR "COPD" OR "chronic respiratory disease" OR "asthma" OR "chronic kidney disease" OR "CKD" OR "renal insufficiency" OR "liver disease" OR "cirrhosis" OR "hepatitis" OR "digestive disease" OR "IBD" OR "Crohn" OR "celiac" OR "arthritis" OR "fibromyalgia" OR "axial spondyloarthritis" OR "musculoskeletal" OR "Parkinson" OR "Alzheimer" OR "neurodegenerative") AND ("care" OR "management" OR "nursing" OR "patient education" OR "self-management" OR "decision support" OR "health education")

**CNKI（n=53）**

SU=("large language model" OR "LLM" OR "ChatGPT" OR "generative AI" OR "大语言模型" OR "生成式人工智能") AND SU=("chronic disease" OR "chronic diseases" OR "chronic disease management" OR "chronic disease nursing" OR "diabetes" OR "糖尿病" OR "hypertension" OR "高血压" OR "cardiovascular disease" OR "心血管疾病" OR "冠心病" OR "stroke" OR "脑卒中" OR "cancer" OR "肿瘤" OR "恶性肿瘤" OR "COPD" OR "慢性阻塞性肺疾病" OR "chronic respiratory disease" OR "慢性呼吸系统疾病" OR "chronic kidney disease" OR "慢性肾脏病" OR "CKD" OR "liver disease" OR "肝病" OR "肝硬化" OR "肝炎" OR "digestive disease" OR "消化系统疾病" OR "IBD" OR "克罗恩" OR "乳糜泻" OR "arthritis" OR "关节炎" OR "fibromyalgia" OR "纤维肌痛" OR "axial spondyloarthritis" OR "中轴型脊柱关节炎" OR "Parkinson" OR "帕金森" OR "Alzheimer" OR "阿尔茨海默" OR "neurodegenerative" OR "神经退行性") AND SU=("nursing" OR "护理" OR "management" OR "管理" OR "health education" OR "健康教育" OR "self-management" OR "自我管理" OR "decision support" OR "决策支持")

**Wanfang Data（n=44）**

主题/关键词: ("large language model" OR "LLM" OR "ChatGPT" OR "generative AI" OR "大语言模型" OR "生成式人工智能") AND 主题/关键词: ("chronic disease" OR "慢性病" OR "chronic disease management" OR "慢性病管理" OR "chronic disease nursing" OR "慢性病护理" OR "diabetes" OR "糖尿病" OR "hypertension" OR "高血压" OR "cardiovascular disease" OR "心血管疾病" OR "stroke" OR "脑卒中" OR "cancer" OR "肿瘤" OR "癌症" OR "COPD" OR "慢性阻塞性肺疾病" OR "chronic respiratory disease" OR "chronic kidney disease" OR "慢性肾脏病" OR "CKD" OR "liver disease" OR "肝病" OR "肝硬化" OR "肝炎" OR "digestive disease" OR "消化系统疾病" OR "arthritis" OR "关节炎" OR "fibromyalgia" OR "纤维肌痛" OR "Parkinson" OR "帕金森" OR "Alzheimer" OR "阿尔茨海默") AND 主题/关键词: ("nursing" OR "护理" OR "management" OR "管理" OR "health education" OR "健康教育" OR "self-management" OR "自我管理" OR "decision support" OR "决策支持")

**VIP Database（n=31）**

标题/关键词 = ("large language model" OR "LLM" OR "ChatGPT" OR "generative AI" OR "大语言模型" OR "生成式人工智能") AND 标题/关键词 = ("chronic disease" OR "慢性病" OR "chronic disease management" OR "慢性病管理" OR "chronic disease nursing" OR "慢性病护理" OR "diabetes" OR "糖尿病" OR "hypertension" OR "高血压" OR "cardiovascular disease" OR "心血管疾病" OR "stroke" OR "脑卒中" OR "cancer" OR "肿瘤" OR "癌症" OR "COPD" OR "慢性阻塞性肺疾病" OR "chronic respiratory disease" OR "chronic kidney disease" OR "慢性肾脏病" OR "CKD" OR "liver disease" OR "肝病" OR "肝硬化" OR "肝炎" OR "digestive disease" OR "消化系统疾病" OR "arthritis" OR "关节炎" OR "fibromyalgia" OR "纤维肌痛" OR "Parkinson" OR "帕金森" OR "Alzheimer" OR "阿尔茨海默") AND 标题/关键词 = ("nursing" OR "护理" OR "management" OR "管理" OR "health education" OR "健康教育" OR "self-management" OR "自我管理" OR "decision support" OR "决策支持")

**Chinese Biomedical Literature Database（n=77）**

("large language model"[常用字段] OR "LLM"[常用字段] OR "ChatGPT"[常用字段] OR "generative AI"[常用字段] OR "大语言模型"[常用字段] OR "生成式人工智能"[常用字段])

AND ("chronic disease"[常用字段] OR "慢性病"[常用字段] OR "chronic disease management"[常用字段] OR "慢性病管理"[常用字段] OR "chronic disease nursing"[常用字段] OR "慢性病护理"[常用字段] OR "diabetes"[常用字段] OR "糖尿病"[常用字段] OR "hypertension"[常用字段] OR "高血压"[常用字段] OR "cardiovascular disease"[常用字段] OR "心血管疾病"[常用字段] OR "冠心病"[常用字段] OR "stroke"[常用字段] OR "脑卒中"[常用字段] OR "cancer"[常用字段] OR "肿瘤"[常用字段] OR "癌症"[常用字段] OR "COPD"[常用字段] OR "慢性阻塞性肺疾病"[常用字段] OR "chronic respiratory disease"[常用字段] OR "chronic kidney disease"[常用字段] OR "慢性肾脏病"[常用字段] OR "CKD"[常用字段] OR "liver disease"[常用字段] OR "肝病"[常用字段] OR "肝硬化"[常用字段] OR "肝炎"[常用字段] OR "digestive disease"[常用字段] OR "消化系统疾病"[常用字段] OR "IBD"[常用字段] OR "克罗恩"[常用字段] OR "乳糜泻"[常用字段] OR "arthritis"[常用字段] OR "关节炎"[常用字段] OR "fibromyalgia"[常用字段] OR "纤维肌痛"[常用字段] OR "axial spondyloarthritis"[常用字段] OR "中轴型脊柱关节炎"[常用字段] OR "Parkinson"[常用字段] OR "帕金森"[常用字段] OR "Alzheimer"[常用字段] OR "阿尔茨海默"[常用字段] OR "neurodegenerative"[常用字段] OR "神经退行性"[常用字段]) AND ("nursing"[常用字段] OR "护理"[常用字段] OR "management"[常用字段] OR "管理"[常用字段] OR "health education"[常用字段] OR "健康教育"[常用字段] OR "self-management"[常用字段] OR "自我管理"[常用字段] OR "decision support"[常用字段] OR "决策支持"[常用字段])
