# Supplementary material for: Application of Large Language Models in Chronic Disease Care: Mixed Methods Systematic Review and Thematic Synthesis
Source: J Med Internet Res. 2026 Aug 11;28:e90744. doi: 10.2196/90744 (PMC13460802; doi:10.2196/90744)
Supplement: Supplementary file 2 [file jmir-v28-e90744-s002.docx]

Subject comprehensive coding table

| Included Study (Author, Year) | Verbatim Excerpt (key finding) | Descriptive Code | Descriptive Theme |
| --- | --- | --- | --- |
| Usen A, 2025 | “ChatGPT-4o and Gemini 2.0 demonstrated superior reliability/usability; improved information accessibility.” | Improved reliability and usability of newer LLMs | Application efficacy (clinical decision support) |
| Amidei, 2025 | “High accuracy, comparable to experts; exhibited cross-linguistic adaptability.” | High accuracy and cross-linguistic adaptability | Application efficacy (patient self-assessment) |
| Li, 2025 | “ChatGPT-4.0 achieved the highest accuracy; however, excessive readability levels limited its usability.” | High accuracy but excessive readability | Challenges & limitations (readability mismatch) |
| Giuffrè, 2025 | “RAG-Top10 achieved 91.7% accuracy, reduced hallucinations, and improved guideline adherence.” | RAG improves accuracy and reduces hallucinations | Application efficacy (treatment decision support) |
| Zhang, 2025 | “Diagnostic accuracy improved by 9.59%–22.47%; enhanced patient self-management.” | Fine-tuned model improves diagnostic accuracy | Application efficacy (disease diagnosis support) |
| Mao, 2025 | “KSrisk-GPT identified risk with 95.9% accuracy; improved patient cognition.” | High accuracy in risk factor identification | Application efficacy (disease prevention support) |
| Rullo, 2025 | “Fine-tuning improved accuracy, readability, and professionalism; reduced jargon.” | Fine-tuning improves readability and reduces jargon | Application efficacy (patient health education) |
| Jamil, 2025 | “ChatGPT 4.0 exhibited the best readability and consistency; overall high information accessibility.” | Good readability and consistency | Application efficacy (patient health education) |
| Pawana, 2025 | “Fine-tuned LLaMA 3.2 was optimal for anomaly detection; improved monitoring capabilities.” | Fine-tuned lightweight model effective for anomaly detection | Application efficacy (disease management and control) |
| Kim, 2025 | “Most LLMs achieved >90% accuracy in symptom identification; Llama 3.1 405B performed best.” | High accuracy in symptom detection | Application efficacy (detection of disease symptoms) |
| Zeng, 2025 | “Physicians acknowledged management potential, but concerns regarding liability and misinformation persisted.” | Liability and misinformation concerns | Challenges & limitations (ethical and safety) |
| Healey E, 2025 | “Perfect scores on 9/10 quantitative metrics; high scores (8–10/10) for accuracy, completeness, and safety in qualitative summaries.” | High accuracy, completeness, and safety in CGM data summarization | Application efficacy (data summarization) |
| O’Sullivan JW, 2026 | “Physicians assisted by AMIE were preferred by experts, made fewer errors (24.3% vs. 13.1%), and had fewer omissions (37.4% vs. 17.8%).” | AI assistance reduces errors and omissions | Application efficacy (clinical decision support) |
| Furtado V, 2025 | “Personalization increased engagement by 26% and quadrupled message length; no hallucinations but general advice required caution.” | Personalization increases engagement; no hallucinations | Application efficacy (patient management support) |
| Zhang Y, 2026 | “GPT-5 had the highest GQS score; readability varied significantly among models, showing weak correlation with quality.” | High quality but variable readability | Challenges & limitations (quality-readability decoupling) |
| Bertani L, 2025 | “Gemini was optimal for accuracy, clarity, and readability; however, all models exhibited 13–24% misinformation.” | High accuracy and clarity but notable misinformation | Challenges & limitations (misinformation risk) |
| Carl N, 2025 | “UroBot was superior in correctness (73% vs. 50%), source attribution (74% vs. 30%), and verifiability (84% vs. 35%); physicians trusted it more.” | RAG improves source attribution and verifiability, building trust | Application efficacy (clinical decision support) |
| Alredaini R, 2026 | “GPT-4.1 performed best at 30/60 minutes; LLaMA-7B at 90 minutes; LLMs outperformed other models.” | LLMs outperform traditional models in glucose prediction | Application efficacy (disease prediction) |
