# Supplementary material for: Application of Large Language Models in Chronic Disease Care: Mixed Methods Systematic Review and Thematic Synthesis
Source: J Med Internet Res. 2026 Aug 11;28:e90744. doi: 10.2196/90744 (PMC13460802; doi:10.2196/90744)
Supplement: Supplementary file 3 [file jmir-v28-e90744-s003.docx]

**PRISMA** **2020** **expanded** **checklist**

Note: This expanded checklist details elements recommended for reporting for each item in the[PRISMA 2020 statement](https://www.bmj.com/content/372/bmj.n71). Non-italicized elements are considered ‘essential’ and should be reported in the main report or as supplementary material for all systematic reviews (except for those preceded by “If…”, which should only be reported where applicable). Elements written in italics are

‘additional’, and while not essential, provide supplementary information that may enhance the completeness and usability of systematic review reports. Note that elements presented here are an

abridged version of those presented in the[explanation and elaboration paper](https://www.bmj.com/content/372/bmj.n160)(BMJ 2021;372:n160), with references and some examples removed. Consulting the explanation and elaboration paper is recommended if further clarity or information is required.

| **Section** **and** **Topic** | **Item** **#** | | |   **Items** **and** **elements** **recommended** **for** **reporting** | **Page** | **Notes** |  |  |
| --- | --- | --- | --- | --- | --- | --- | --- | --- |
| **TITLE** | | | | | **1** |  |  |  |
| **TITLE** |   **1** | | | **Item:** **Identify** **the** **report** **as** **a** **systematic** **review.**  Elements:  • Identify the report as a systematic review in the title.  • Report an informative title that provides key information about the main objective or question the review addresses (e.g. the population(s) and intervention(s) the review addresses).  • *Consider* *providing* *additional* *information* *in* *the* *title,* *such* *as* *the* *method* *of* *analysis* *used,* *the* *designs* *of* *included* *studies,* *or* *an* *indication* *that* *the* *review* *is* *an* *update* *of* *an* *existing* *review,* *or* *a* *continually* *updated* *(“living”)* *systematic* *review.* | 1 |  |  |  |
| **ABSTRACT** | | | | | **1** |  |  |  |
| **ABSTRACT** | **2** | | | **Item:** **See** **the** **PRISMA** **2020** **for** **Abstracts** **checklist.**  Elements:  • Report an abstract addressing each item in the PRISMA 2020 for Abstracts checklist. | 1 |  |  |  |
| **INTRODUCTION** | | | | | **2** |  |  |  |
| **RATIONALE** | **3** | | | **Item:** **Describe** **the** **rationale** **for** **the** **review** **in** **the** **context** **of** **existing** **knowledge.**  Elements:  • Describe the current state of knowledge and its uncertainties.  • Articulate why it is important to do the review.  • If other systematic reviews addressing the same (or a largely similar) question are available, explain why the current review was considered necessary. If the review is an update or replication of a particular systematic review, indicate this and cite the previous review.  • If the review examines the effects of interventions, also briefly describe how the intervention(s) examined might work.  • *If* *there* *is* *complexity* *in* *the* *intervention* *or* *context* *of* *its* *delivery* *(or* *both)* *(e.g.* *multi-component* *interventions,* *equity* *considerations),* *consider* *presenting* *a* *logic* *model* *to* *visually* *display* *the* *hypothesised* *relationship* *between* *intervention* *components* *and* *outcomes.* | 2-3 |  |  |  |
| **OBJECTIVES** | **4** | | | **Item:** **Provide** **an** **explicit** **statement** **of** **the** **objective(s)** **or** **question(s)** **the** **review** **addresses.**  Elements:  • Provide an explicit statement of all objective(s) or question(s) the review addresses, expressed in terms of a relevant question formulation framework.  • If the purpose is to evaluate the effects of interventions, use the Population, Intervention, Comparator, Outcome (PICO) framework or one of its variants, to state the comparisons that will be made. | 3 |  |  |  |
| **METHODS** | | | | | **4** |  |  |  |
| **ELIGIBILITY** **CRITERIA** | **5** | | | **Item:** **Specify** **the** **inclusion** **and** **exclusion** **criteria** **for** **the** **review** **and** **how** **studies** **were** **grouped** **for** **the** **syntheses.**  Elements:  • Specify all study characteristics used to decide whether a study was eligible for inclusion in the review, that is, components described in the PICO  framework or one of its variants, and other characteristics, such as eligible study design(s) and setting(s), and minimum duration of follow-up.  • Specify eligibility criteria with regard to report characteristics, such as year of dissemination, language, and report status (e.g. whether reports, such as unpublished manuscripts and conference abstracts, were eligible for inclusion).  • Clearly indicate if studies were ineligible because the outcomes of interest were not measured, or ineligible because the results for the outcome of interest were not reported.  • Specify any groups used in the synthesis (e.g. intervention, outcome and population groups) and link these to the comparisons specified in the objectives(item #4).  • *Consider* *providing* *rationales* *for* *any* *notable* *restrictions* *to* *study* *eligibility.* | 4-5 |  |  |  |
| **INFORMATION** **SOURCES** | **6** | | **Item:** **Specify** **all** **databases,** **registers,** **websites,** **organisations,** **reference** **lists** **and** **other** **sources** **searched** **or** **consulted** **to** **identify** **studies.** **Specify** **the** **date** **when** **each** **source** **was** **last** **searched** **or** **consulted.**  Elements:  • Specify the date when each source (e.g. database, register, website, organisation) was last searched or consulted.  • If bibliographic databases were searched, specify for each database its name (e.g. MEDLINE, CINAHL), the interface or platform through which the database was searched (e.g. Ovid, EBSCOhost), and the dates of coverage (where this information is provided).  • If study registers, regulatory databases and other online repositories were searched, specify the name of each source and any date restrictions that were applied.  • If websites, search engines or other online sources were browsed or searched, specify the name and URL of each source.  • If organisations or manufacturers were contacted to identify studies, specify the name of each source.  • If individuals were contacted to identify studies, specify the types of individuals contacted (e.g. authors of studies included in the review or researchers with expertise in the area).  • If reference lists were examined, specify the types of references examined (e.g. references cited in study reports included in the systematic review, or references cited in systematic review reports on the same or similar topic).  • If cited or citing reference searches (also called backward and forward citation searching) were conducted, specify the bibliographic details of the reports to which citation searching was applied, the citation index or platform used (e.g. Web of Science), and the date the citation searching was done.  • If journals or conference proceedings were consulted, specify of the names of each source, the dates covered and how they were searched (e.g. handsearching or browsing online). | | 4 |  |  |  |
| **SEARCH** **STRATEGY** | **7** | | **Item:** **Present** **the** **full** **search** **strategies** **for** **all** **databases,** **registers** **and** **websites,** **including** **any** **filters** **and** **limits** **used.**  Element:  • Provide the full line by line search strategy as run in each database with a sophisticated interface (such as Ovid), or the sequence of terms that were used to search simpler interfaces, such as search engines or websites.  • Describe any limits applied to the search strategy (e.g. date or language) and justify these by linking back to the review’s eligibility criteria.  • If published approaches, including search filters designed to retrieve specific types of records or search strategies from other systematic reviews, were used, cite them. If published approaches were adapted, for example if search filters are amended, note the changes made.  • If natural language processing or text frequency analysis tools were used to identify or refine keywords, synonyms or subject indexing terms to use in the search strategy, specify the tool(s) used.  • If a tool was used to automatically translate search strings for one database to another, specify the tool used.  • If the search strategy was validated, for example by evaluating whether it could identify a set of clearly eligible studies, report the validation process used and specify which studies were included in the validation set.  • If the search strategy was peer reviewed, report the peer review process used and specify any tool used such as the Peer Review of Electronic Search Strategies (PRESS) checklist.  • If the search strategy structure adopted was not based on a PICO-style approach, describe the final conceptual structure and any explorations that were undertaken to achieve it. | | 4 |  |  |  |
| **SELECTION** **PROCESS** | | **8** | **Item:** **Specify** **the** **methods** **used** **to** **decide** **whether** **a** **study** **met** **the** **inclusion** **criteria** **of** **the** **review,** **including** **how** **many** **reviewers** **screened** **each** **record** **and** **each** **report** **retrieved,** **whether** **they** **worked** **independently,** **and** **if** **applicable,** **details** **of** **automation** **tools** **used** **in** **the** **process.**  Elements:  *Recommendations* *for* *reporting* *regardless* *of* *the* *selection* *processes* *used:*  • Report how many reviewers screened each record (title/abstract) and each report retrieved, whether multiple reviewers worked independently at each stage of screening or not, and any processes used to resolve disagreements between screeners.  • Report any processes used to obtain or confirm relevant information from study investigators.  • If abstracts or articles required translation into another language to determine their eligibility, report how these were translated.  *Recommendations* *for* *reporting* *in* *systematic* *reviews* *using* *automation* *tools* *in* *the* *selection* *process:*  • Report how automation tools were integrated within the overall study selection process.  • If an externally derived machine learning classifier was applied (e.g. Cochrane RCT Classifier), either to eliminate records or to replace a single screener, include a reference or URL to the version used. If the classifier was used to eliminate records *before* *screening*, report the number eliminated in the  PRISMA flow diagram as ‘Records marked as ineligible by automation tools’ .  • If an internally derived machine learning classifier was used to assist with the screening process, identify the software/classifier and version, describe how it was used (e.g. to remove records or replace a single screener) and trained (if relevant), and what internal or external validation was done to understand the risk of missed studies or incorrect classifications.  • If machine learning algorithms were used to prioritise screening (whereby unscreened records are continually re-ordered based on screening decisions), state the software used and provide details of any screening rules applied.  *Recommendations* *for* *reporting* *in* *systematic* *reviews* *using* *crowdsourcing* *or* *previous* *‘known’* *assessments* *in* *the* *selection* *process:*  • If crowdsourcing was used to screen records, provide details of the platform used and specify how it was integrated within the overall study selection process.  • If datasets of already-screened records were used to eliminate records retrieved by the search from further consideration, briefly describe the derivation of these datasets. | | 5 |  |  |  |
| **DATA** **COLLECTION** **PROCESS** | | **9** | **Item:** **Specify** **the** **methods** **used** **to** **collect** **data** **from** **reports,** **including** **how** **many** **reviewers** **collected** **data** **from** **each** **report,** **whether** **they** **worked** **independently,** **any** **processes** **for** **obtaining** **or** **confirming** **data** **from** **study** **investigators,** **and** **if** **applicable,** **details** **of** **automation** **tools** **used** **in** **the** **process.**  Elements:  • Report how many reviewers collected data from each report, whether multiple reviewers worked independently or not, and any processes used to resolve disagreements between data collectors.  • Report any processes used to obtain or confirm relevant data from study investigators.  • If any automation tools were used to collect data, report how the tool was used, how the tool was trained, and what internal or external validation was done to understand the risk of incorrect extractions.  • If articles required translation into another language to enable data collection, report how these articles were translated.  • If any software was used to extract data from figures, specify the software used.  • If any decision rules were used to select data from multiple reports corresponding to a study, and any steps were taken to resolve inconsistencies across reports, report the rules and steps used. | | 5 |  |  |  |
| **DATA** **ITEMS**  **(outcomes)**  •  •  •  • | | **10a** | **Item:** **List** **and** **define** **all** **outcomes** **for** **which** **data** **were** **sought.** **Specify** **whether** **all** **results** **that** **were** **compatible** **with** **each** **outcome** **domain** **in** **each** **study** **were** **sought** **(e.g.** **for** **all** **measures,** **time** **points,** **analyses),** **and** **if** **not,** **the** **methods** **used** **to** **decide** **which** **results** **to** **collect.**  Elements:  • List and define the outcome domains and time frame of measurement for which data were sought.  • Specify whether all results that were compatible with each outcome domain in each study were sought, and if not, what process was used to select results within eligible domains.  • If any changes were made to the inclusion or definition of the outcome domains, or to the importance given to them in the review, specify the changes, along with a rationale.  • If any changes were made to the processes used to select results within eligible outcome domains, specify the changes , along with a rationale.  • Consider specifying which outcome domains were considered the most important for interpreting the review’s conclusions and provide rationale for the labelling (e.g. “a recent core outcome set identified the outcomes labelled ‘critical’ as being the most important to patients”). | | 5 |  |  |  |
| **DATA** **ITEMS** **(other** **variables)** | | **10b** | **Item:** **List** **and** **define** **all** **other** **variables** **for** **which** **data** **were** **sought** **(e.g.** **participant** **and** **intervention** **characteristics,** **funding** **sources).** **Describe** **any** **assumptions** **made** **about** **any** **missing** **or** **unclear** **information.**  Elements:  • List and define all other variables for which data were sought (e.g. participant and intervention characteristics, funding sources).  • Describe any assumptions made about any missing or unclear information from the studies.  • If a tool was used to inform which data items to collect, cite the tool used. | | 5 |  | |  |
| **STUDY** **RISK** **OF** **BIAS** **ASSESSMENT** | | **11** | **Item:** **Specify** **the** **methods** **used** **to** **assess** **risk** **of** **bias** **in** **the** **included** **studies,** **including** **details** **of** **the** **tool(s)** **used,** **how** **many** **reviewers** **assessed** **each** **study** **and** **whether** **they** **worked** **independently,** **and** **if** **applicable,** **details** **of** **automation** **tools** **used** **in** **the** **process.**  Elements:  • Specify the tool(s) (and version) used to assess risk of bias in the included studies.  • Specify the methodological domains/components/items of the risk of bias tool(s) used.  • Report whether an overall risk of bias judgement that summarised across domains/components/items was made, and if so, what rules were used to reach an overall judgement.  • If any adaptations to an existing tool to assess risk of bias in studies were made, specify the adaptations.  • If a new risk of bias tool was developed for use in the review, describe the content of the tool and make it publicly accessible.  • Report how many reviewers assessed risk of bias in each study, whether multiple reviewers worked independently, and any processes used to resolve disagreements between assessors.  • Report any processes used to obtain or confirm relevant information from study investigators.  • If an automation tool was used to assess risk of bias, report how the automation tool was used, how the tool was trained, and details on the tool’s performance and internal validation. | | 5 |  | |  |
| **EFFECT** **MEASURES** | | **12** | **Item:** **Specify** **for** **each** **outcome** **the** **effect** **measure(s)** **(e.g.** **risk** **ratio,** **mean** **difference)** **used** **in** **the** **synthesis** **or** **presentation** **of** **results.**  Elements:  • Specify for each outcome (or type of outcome [e.g. binary, continuous]), the effect measure(s) (e.g. risk ratio, mean difference) used in the synthesis or presentation of results.  • State any thresholds (or ranges) used to interpret the size of effect (e.g. minimally important difference; ranges for no/trivial, small, moderate and large effects) and the rationale for these thresholds.  • If synthesized results were re-expressed to a different effect measure, report the method used to re-express results (e.g. meta-analysing risk ratios and computing an absolute risk reduction based on an assumed comparator risk).  • *Consider* *providing* *justification* *for* *the* *choice* *of* *effect* *measure.* | | 5-6 | This review did not conduct a meta-analysis or quantitative statistical synthesis, and therefore no standardized effect sizes (such as risk ratios or mean differences) were used. In the "Results of Individual Studies" section, we presented descriptive results from each study—such as accuracy, F1 scores, and readability scores—in narrative form and tables, without combining or standardizing effect sizes. This approach has been explained in the "Synthesis Methods" section (see pages 5–6). | |  |
| **SYNTHESIS** **METHODS** **(eligibility** **for**  **synthesis)** | | **13a** | **Item:** **Describe** **the** **processes** **used** **to** **decide** **which** **studies** **were** **eligible** **for** **each** **synthesis** **(e.g.** **tabulating** **the** **study** **intervention** **characteristics** **and** **comparing** **against** **the** **planned** **groups** **for** **each** **synthesis** **(item** **#5)).**  Element:  • Describe the processes used to decide which studies were eligible for each synthesis. | | 5-6 |  | |  |
| **SYNTHESIS** **METHODS** **(preparing** **for**  **synthesis)** | | **13b** | **Item:** **Describe** **any** **methods** **required** **to** **prepare** **the** **data** **for** **presentation** **or** **synthesis,** **such** **as** **handling** **of** **missing** **summary** **statistics,** **or** **data** **conversions.**  Element:  • Report any methods required to prepare the data collected from studies for presentation or synthesis, such as handling of missing summary statistics, or data conversions. | | Not applicable. | This review employed a thematic synthesis approach, and all included studies were narrative text reports without quantitative data to be pooled. Summary statistics for all included studies have been fully reported in the original texts, making additional data transformation or imputation of missing statistics unnecessary. Therefore, this item is not applicable. | |  |
| **SYNTHESIS** **METHODS** **(tabulation** **and**  **graphical** **methods)** | | **13c** | **Item:** **Describe** **any** **methods** **used** **to** **tabulate** **or** **visually** **display** **results** **of** **individual** **studies** **and** **syntheses.**  Elements:  • Report chosen tabular structure(s) used to display results of individual studies and syntheses, along with details of the data presented.  • Report chosen graphical methods used to visually display results of individual studies and syntheses.  • *If* *studies* *are* *ordered* *or* *grouped* *within* *tables* *or* *graphs* *based* *on* *study* *characteristics* *(e.g.* *by* *size* *of* *the* *study* *effect,* *year* *of* *publication),* *consider* *reporting* *the* *basis* *for* *the* *chosen* *ordering/grouping.*  • *If* *non-standard* *graphs* *were* *used,* *consider* *reporting* *the* *rationale* *for* *selecting* *the* *chosen* *graph.* | | 5-6 |  | |  |
| **SYNTHESIS** **METHODS** **(statistical** **synthesis**  **methods)** | | **13d** | **Item:** **Describe** **any** **methods** **used** **to** **synthesize** **results** **and** **provide** **a** **rationale** **for** **the** **choice(s).** **If** **meta-analysis** **was** **performed,** **describe** **the** **model(s),** **method(s)** **to** **identify** **the** **presence** **and** **extent** **of** **statistical** **heterogeneity,** **and** **software** **package(s)** **used.**  Elements:  • If statistical synthesis methods were used, reference the software, packages and version numbers used to implement synthesis methods.  • If it was not possible to conduct a meta-analysis, describe and justify the synthesis methods or summary approach used.  • If meta-analysis was done, specify:  o the meta-analysis model (fixed-effect, fixed-effects or random-effects) and provide rationale for the selected model.  o the method used (e.g. Mantel-Haenszel, inverse-variance).  o any methods used to identify or quantify statistical heterogeneity (e.g. visual inspection of results, a formal statistical test for heterogeneity, heterogeneity variance (τ2 ), inconsistency (e.g. I^2^), and prediction intervals).  • If a random-effects meta-analysis model was used:  o specify the between-study (heterogeneity) variance estimator used (e.g. DerSimonian and Laird, restricted maximum likelihood (REML)).  o specify the method used to calculate the confidence interval for the summary effect (e.g. Wald-type confidence interval, Hartung-Knapp-Sidik- Jonkman).  o *consider* *specifying* *other* *details* *about* *the* *methods* *used,* *such* *as* *the* *method* *for* *calculating* *confidence* *limits* *for* *the* *heterogeneity* *variance.*  • If a Bayesian approach to meta-analysis was used, describe the prior distributions about quantities of interest (e.g. intervention effect being analysed, amount of heterogeneity in results across studies).  • If multiple effect estimates from a study were included in a meta-analysis, describe the method(s) used to model or account for the statistical dependency (e.g. multivariate meta-analysis, multilevel models or robust variance estimation).  • If a planned synthesis was not considered possible or appropriate, report this and the reason for that decision. | | 5-6 |  | |  |
| **SYNTHESIS** **METHODS** **(methods** **to** **explore**  **heterogeneity)**  •  • | | **13e** | **Item:** **Describe** **any** **methods** **used** **to** **explore** **possible** **causes** **of** **heterogeneity** **among** **study** **results** **(e.g.** **subgroup** **analysis,** **meta-regression).**  Elements:  • If methods were used to explore possible causes of statistical heterogeneity, specify the method used (e.g. subgroup analysis , meta-regression).  • If subgroup analysis or meta-regression was performed, specify for each:  o which factors were explored, levels of those factors, and which direction of effect modification was expected and why (where possible).  o whether analyses were conducted using study-level variables (i.e. where each study is included in one subgroup only), within-study contrasts (i.e. where data on subsets of participants within a study are available, allowing the study to be included in more than one subgroup), or some  combination of the above.  o how subgroup effects were compared (e.g. statistical test for interaction for subgroup analyses).  If other methods were used to explore heterogeneity because data were not amenable to meta-analysis of effect estimates (e.g. structuring tables to examine variation in results across studies based on subpopulation), describe the methods used, along with the factors and levels.  If any analyses used to explore heterogeneity were not pre-specified, identify them as such. | | Not applicable. | This review did not conduct a meta-analysis, so no formal test for statistical heterogeneity was performed (such as subgroup analysis or meta-regression).This review did not conduct a meta-analysis, so no formal test for statistical heterogeneity was performed (such as subgroup analysis or meta-regression). Although we provided a narrative comparison of differences among different study designs and disease types in the discussion section, no formal heterogeneity exploration plan was pre-defined in the methods section. Therefore, this entry is not applicable. Although we provided a narrative comparison of differences among different study designs and disease types in the discussion section, no formal heterogeneity exploration plan was pre-defined in the methods section. Therefore, this entry is not applicable. | |  |
| **SYNTHESIS** **METHODS** **(sensitivity** **analyses)** | | **13f** | **Item:** **Describe** **any** **sensitivity** **analyses** **conducted** **to** **assess** **robustness** **of** **the** **synthesized** **results.**  Elements:  • If sensitivity analyses were performed, provide details of each analysis (e.g. removal of studies at high risk of bias, use of an alternative meta-analysis model).  • If any sensitivity analyses were not pre-specified, identify them as such. | | Not applicable. | This review did not conduct sensitivity analysis.This review did not conduct sensitivity analysis. All 20 included studies participated in a unified thematic synthesis, and no studies were excluded or grouped for comparison. All 20 included studies participated in a unified thematic synthesis, and no studies were excluded or grouped for comparison. All studies were rated as moderate or high quality, so there was no need to exclude low-quality studies to test the robustness of All studies were rated as medium or high quality, so there was no needthe results. to exclude low-quality studies to test the robustness of the results. This item is not applicable. | |  |
| **REPORTING** **BIAS** **ASSESSMENT** | | **14** | **Item:** **Describe** **any** **methods** **used** **to** **assess** **risk** **of** **bias** **due** **to** **missing** **results** **in** **a** **synthesis** **(arising** **from** **reporting** **biases).**  Elements:  • Specify the methods (tool, graphical, statistical or other) used to assess the risk of bias due to missing results in a synthesis (arising from reporting biases).  • If risk of bias due to missing results was assessed using an existing tool, specify the methodological components/domains/items of the tool, and the process used to reach a judgement of overall risk of bias.  • If any adaptations to an existing tool to assess risk of bias due to missing results were made, specify the adaptations.  • If a new tool to assess risk of bias due to missing results was developed for use in the review, describe the content of the tool and make it publicly accessible.  • Report how many reviewers assessed risk of bias due to missing results in a synthesis, whether multiple reviewers worked independently, and any processes used to resolve disagreements between assessors.  • Report any processes used to obtain or confirm relevant information from study investigators.  • If an automation tool was used to assess risk of bias due to missing results, report how the automation tool was used, how the tool was trained, and details on the tool’s performance and internal validation. | | 5 |  | |  |
| **CERTAINTY**  **ASSESSMENT** | | **15** | **Item:** **Describe** **any** **methods** **used** **to** **assess** **certainty** **(or** **confidence)** **in** **the** **body** **of** **evidence** **for** **an** **outcome.**  Elements:  • Specify the tool or system (and version) used to assess certainty (or confidence) in the body of evidence.  • Report the factors considered (e.g. precision of the effect estimate, consistency of findings across studies) and the criteria used to assess each factor when assessing certainty in the body of evidence.  • Describe the decision rules used to arrive at an overall judgement of the level of certainty, together with the intended interpretation (or definition) of each level of certainty.  • If applicable, report any review-specific considerations for assessing certainty, such as thresholds used to assess imprecision and ranges of magnitude of effect that might be considered trivial, moderate or large, and the rationale for these thresholds and ranges (item #12).  • If any adaptations to an existing tool or system to assess certainty were made, specify the adaptations.  • Report how many reviewers assessed certainty in the body of evidence for an outcome, whether multiple reviewers worked independently, and any processes used to resolve disagreements between assessors.  • Report any processes used to obtain or confirm relevant information from investigators.  • If an automation tool was used to support the assessment of certainty, report how the automation tool was used, how the tool was trained, and details on the tool’s performance and internal validation.  • Describe methods for reporting the results of assessments of certainty, such as the use of Summary of Findings tables.  • If standard phrases that incorporate the certainty of evidence were used (e.g. “hip protectors *probably* reduce the risk of hip fracture slightly”), report the intended interpretation of each phrase and the reference for the source guidance. | | Not applicable. | This review did not use formal tools such as GRADE or CERQual to assess the confidence level of the evidence body. The main reason for this is that the included research designs are highly heterogeneous (covering quantitative, qualitative, and mixed-methods), and the review is mainly focused on thematic synthesis, which is not suitable for using assessment tools like GRADE that are mainly designed for evaluating the effect sizes of intervention studies. We have provided a descriptive account of the limitations of the evidence in the "Limitations" section of the discussion. | |  |
| **RESULTS** | | | | | 6 |  | | |
| **STUDY** **SELECTION** **(flow** **of** **studies)** | | **16a** | | **Item:** **Describe** **the** **results** **of** **the** **search** **and** **selection** **process,** **from** **the** **number** **of** **records** **identified** **in** **the** **search** **to** **the** **number** **of** **studies** **included** **in** **the** **review,** **ideally** **using** **a** **flow** **diagram.**  Elements:  • Report, ideally using a flow diagram, the number of: records identified; records excluded before screening; records screened; records excluded after  screening titles or titles and abstracts; reports retrieved for detailed evaluation; potentially eligible reports that were not retrievable; retrieved reports that did not meet inclusion criteria and the primary reasons for exclusion; and the number of studies and reports included in the review. If applicable, also  report the number of ongoing studies and associated reports identified.  • If the review is an update of a previous review, report results of the search and selection process for the current review and specify the number of studies included in the previous review.  • If applicable, indicate in the PRISMA flow diagram how many records were excluded by a human and how many by automation tools. | 6-7 |  | | |
| **STUDY** **SELECTION**  **(excluded** **studies)** | | **16b** | | **Item:** **Cite** **studies** **that** **might** **appear** **to** **meet** **the** **inclusion** **criteria,** **but** **which** **were** **excluded,** **and** **explain** **why** **they** **were** **excluded.**  Element:  • Cite studies that might appear to meet the inclusion criteria, but which were excluded, and explain why they were excluded. | 6-7 |  | | |
| **STUDY**  **CHARACTERISTICS** | | **17** | | **Item:** **Cite** **each** **included** **study** **and** **present** **its** **characteristics.**  Elements:  • Cite each included study.  • Present the key characteristics of each study in a table or figure (considering a format that will facilitate comparison of characteristics across the studies).  • *If* *the* *review* *examines* *the* *effects* *of* *interventions,* *consider* *presenting* *an* *additional* *table* *that* *summarises* *the* *intervention* *details* *for* *each* *study.* | 7-8 |  | | |
| **RISK** **OF** **BIAS** **IN** **STUDIES** | | **18** | | **Item:** **Present** **assessments** **of** **risk** **of** **bias** **for** **each** **included** **study.**  Elements:  • Present tables or figures indicating for each study the risk of bias in each domain/component/item assessed (e.g. blinding of outcome assessors, missing outcome data) and overall study-level risk of bias.  • Present justification for each risk of bias judgement, for example in the form of relevant quotations from reports of included studies.  • *If* *assessments* *of* *risk* *of* *bias* *were* *done* *for* *specific* *outcomes* *or* *results* *in* *each* *study,* *consider* *displaying* *risk* *of* *bias* *judgements* *on* *a* *forest* *plot,* *next* *to* *the* *study* *results.* | 13-14 |  | | |
| **RESULTS** **OF**  **INDIVIDUAL** **STUDIES** | | **19** | | **Item:** **For** **all** **outcomes,** **present,** **for** **each** **study:** **(a)** **summary** **statistics** **for** **each** **group** **(where** **appropriate)** **and** **(b)** **an** **effect** **estimate** **and** **its** **precision** **(e.g.** **confidence/credible** **interval),** **ideally** **using** **structured** **tables** **or** **plots.**  Elements:  • For all outcomes, irrespective of whether statistical synthesis was undertaken, present for each study summary statistics for each group (where  appropriate). For dichotomous outcomes, report the number of participants with and without the events for each group; or the number with the event and the total for each group (e.g. 12/45). For continuous outcomes, report the mean, standard deviation and sample size of each group.  • For all outcomes, irrespective of whether statistical synthesis was undertaken, present for each study an effect estimate and its precision (e.g. standard error or 95% confidence/credible interval). For example, for time-to-event outcomes, present a hazard ratio and its confidence interval.  • If study-level data is presented visually or reported in the text (or both), also present a tabular display of the results.  • If results were obtained from multiple data sources (e.g. journal article, study register entry, clinical study report, correspondence with authors), report the source of the data.  • If applicable, indicate which results were not reported directly and had to be computed or estimated from other information. | 8-13 |  | | |
| **RESULTS** **OF**  **SYNTHESES** | | **20a** | | **Item:** **For** **each** **synthesis,** **briefly** **summarise** **the** **characteristics** **and** **risk** **of** **bias** **among** **contributing** **studies.**  Elements: | 14-15 |  | | |

| **(characteristics** **of**  **contributing** **studies)** |  | | • Provide a brief summary of the characteristics and risk of bias among studies contributing to each synthesis (meta-analysis or other). The summary should focus only on study characteristics that help in interpreting the results (especially those that suggest the evidence addresses only a restricted part of the  review question, or indirectly addresses the question).  • Indicate which studies were included in each synthesis (e.g. by listing each study in a forest plot or table or citing studies in the text). |  |  | |
| --- | --- | --- | --- | --- | --- | --- |
| **RESULTS** **OF**  **SYNTHESES** **(results** **of** **statistical** **syntheses)** | **20b** | | **Item:** **Present** **results** **of** **all** **statistical** **syntheses** **conducted.** **If** **meta-analysis** **was** **done,** **present** **for** **each** **the** **summary** **estimate** **and** **its** **precision** **(e.g.** **confidence/credible** **interval)** **and** **measures** **of** **statistical** **heterogeneity.** **If** **comparing** **groups,** **describe** **the** **direction** **of** **the** **effect.**  Elements:  • Report results of all statistical syntheses described in the protocol and all syntheses conducted that were not pre-specified.  • If meta-analysis was conducted, report for each:  o the summary estimate and its precision (e.g. standard error or 95% confidence/credible interval)  o measures of statistical heterogeneity (e.g. τ 2 , I^2^ , prediction interval)  • If other statistical synthesis methods were used (e.g. summarising effect estimates, combining P values), report the synthesized result and a measure of precision (or equivalent information, for example, the number of studies and total sample size).  • If the statistical synthesis method does not yield an estimate of effect (e.g. as is the case when P values are combined), report the relevant statistics (e.g. P value from the statistical test), along with an interpretation of the result that is consistent with the question addressed by the synthesis method.  • If comparing groups, describe the direction of effect (e.g. fewer events in the intervention group, or higher pain in the comparator group).  • If synthesising mean differences, specify for each synthesis, where applicable, the unit of measurement (e.g. kilograms or pounds for weight), the upper and lower limits of the measurement scale (e.g. anchors range from 0 to 10), direction of benefit (e.g. higher scores denote higher severity of pain), and the minimally important difference, if known. If synthesising standardised mean differences, and the effect estimate is being re-expressed to a particular instrument, details of the instrument, as per the mean difference, should be reported. | 14-15 |  | |
| **RESULTS** **OF**  **SYNTHESES** **(results** **of** **investigations** **of**  **heterogeneity)** | **20c** | | **Item:** **Present** **results** **of** **all** **investigations** **of** **possible** **causes** **of** **heterogeneity** **among** **study** **results.**  Elements:  • If investigations of possible causes of heterogeneity were conducted:  o present results regardless of the statistical significance, magnitude, or direction of effect modification.  o identify the studies contributing to each subgroup.  o report results with due consideration to the observational nature of the analysis and risk of confounding due to other factors.  • If subgroup analysis was conducted:  o report for each analysis the exact P value for a test for interaction, as well as, within each subgroup, the summary estimates, their precision (e.g. standard error or 95% confidence/credible interval) and measures of heterogeneity.  o *consider* *presenting* *the* *estimate* *for* *the* *difference* *between* *subgroups* *and* *its* *precision.*  • If meta-regression was conducted:  o report for each analysis the exact P value for the regression coefficient and its precision.  o *consider* *presenting* *a* *meta-regression* *scatterplot* *with* *the* *study* *effect* *estimates* *plotted* *against* *the* *potential* *effect* *modifier.*  • If informal methods (i.e. those that do not involve a formal statistical test) were used to investigate heterogeneity, describe the results observed. | 14-15 |  | |
| **RESULTS** **OF**  **SYNTHESES** **(results** **of** **sensitivity** **analyses)** | **20d** | | **Item:** **Present** **results** **of** **all** **sensitivity** **analyses** **conducted** **to** **assess** **the** **robustness** **of** **the** **synthesized** **results.**  Elements:  • If any sensitivity analyses were conducted:  o report the results for each sensitivity analysis.  o comment on how robust the main analysis was given the results of all corresponding sensitivity analyses.  o consider presenting results in tables that indicate: (i) the summary effect estimate, a measure of precision (and potentially other relevant  o statistics, for example, I2 statistic) and contributing studies for the original meta-analysis; (ii) the same information for the sensitivity analysis; and (iii) details of the original and sensitivity analysis assumptions.  o consider presenting results of *sensitivity* *analyses* *visually* *using* *forest* *plots.* | 14-15 |  | |
| **REPORTING** **BIASES** | | **21** | **Item:** **Present** **assessments** **of** **risk** **of** **bias** **due** **to** **missing** **results** **(arising** **from** **reporting** **biases)** **for** **each** **synthesis** **assessed.**  Elements:  • Present assessments of risk of bias due to missing results (arising from reporting biases) for each synthesis assessed.  • If a tool was used to assess risk of bias due to missing results in a synthesis, present responses to questions in the tool, judgements about risk of bias and any information used to support such judgements.  • If a funnel plot was generated to evaluate small-study effects (one cause of which is reporting biases), present the plot and specify the effect estimate and measure of precision used in the plot. If a contour-enhanced funnel plot was generated, specify the ‘milestones’ of statistical significance that the plotted  contour lines represent (P = 0.01, 0.05, 0.1, etc.)  • If a test for funnel plot asymmetry was used, report the exact P value observed for the test, and potentially other relevant statistics, for example the standardised normal deviate, from which the P value is derived.  • If any sensitivity analyses seeking to explore the potential impact of missing results on the synthesis were conducted, present results of each analysis (see item #20d), compare them with results of the primary analysis, and report results with due consideration of the limitations of the statistical method.  • *If* *studies* *were* *assessed* *for* *selective* *non-reporting* *of* *results* *by* *comparing* *outcomes* *and* *analyses* *pre-specified* *in* *study* *registers,* *protocols,* *and*  *statistical* *analysis* *plans* *with* *results* *that* *were* *available* *in* *study* *reports,* *consider* *presenting* *a* *matrix* *(with* *rows* *as* *studies* *and* *columns* *as* *syntheses)* *to* *present* *the* *availability* *of* *study* *results.*  • *If* *an* *assessment* *of* *selective* *non-reporting* *of* *results* *reveals* *that* *some* *studies* *are* *missing* *from* *the* *synthesis,* *consider* *displaying* *the* *studies* *with* *missing* *results* *underneath* *a* *forest* *plot* *or* *including* *a* *table* *with* *the* *available* *study* *results.* | 13-14 |  | |
| **CERTAINTY** **OF** **EVIDENCE** | | **22** | **Item:** **Present** **assessments** **of** **certainty** **(or** **confidence)** **in** **the** **body** **of** **evidence** **for** **each** **outcome** **assessed.**  Elements:  • Report the overall level of certainty (or confidence) in the body of evidence for each important outcome.  • Provide an explanation of reasons for rating down (or rating up) the certainty of evidence (e.g. in footnotes to an evidence summary table).  • Communicate certainty in the evidence wherever results are reported (i.e. abstract, evidence summary tables, results, conclusions), using a format appropriate for the section of the review.  • *Consider* *including* *evidence* *summary* *tables,* *such* *as* *GRADE* *Summary* *of* *Findings* *tables.* | Not applicable. | This review did not conduct a formal assessment of the certainty of evidence. The main reason for this is that the included studies have significant heterogeneity in design (covering quantitative, qualitative, and mixed-methods), and the review is mainly focused on thematic synthesis, which is not suitable for using assessment tools such as GRADE that are mainly designed for evaluating the effect sizes of intervention studies. The limitations of the evidence have been presented in the discussion section (page 18, "Limitations") in a narrative manner, but were not standardized and reported using a grading system. | |
| **DISCUSSION** | | | | **16** |  | |
| **DISCUSSION**  **(interpretation)** | | **23a** | **Item:** **Provide** **a** **general** **interpretation** **of** **the** **results** **in** **the** **context** **of** **other** **evidence.**  Element:  • Provide a general interpretation of the results in the context of other evidence. | 16-17 |  | |
| **DISCUSSION**  **(limitations** **of** **evidence)** | | **23b** | **Item:** **Discuss** **any** **limitations** **of** **the** **evidence** **included** **in** **the** **review.**  Element:  • Discuss any limitations of the evidence included in the review. | 18 |  | |
| **DISCUSSION**  **(limitations** **of** **review** **processes)** | | **23c** | **Item:** **Discuss** **any** **limitations** **of** **the** **review** **processes** **used.**  Element:  • Discuss any limitations of the review processes used, and comment on the potential impact of each limitation. | 18 |  | |
| **DISCUSSION**  **(implications)** | | **23d** | **Item:** **Discuss** **implications** **of** **the** **results** **for** **practice,** **policy,** **and** **future** **research.**  Elements:  • Discuss implications of the results for practice and policy.  • Make explicit recommendations for future research. | 18 |  | |
| **OTHER** **INFORMATION** | | | |  |  |  |
| **REGISTRATION** **AND** **PROTOCOL**  **(registration)** | | **24a** | **Item:** **Provide** **registration** **information** **for** **the** **review,** **including** **register** **name** **and** **registration** **number,** **or** **state** **that** **the** **review** **was** **not** **registered.**  Element:  • Provide registration information for the review, including register name and registration number, or state that the review was not registered. | 1、4 |  |  |
| **REGISTRATION** **AND**  **PROTOCOL** **(protocol)** | | **24b** | **Item:** **Indicate** **where** **the** **review** **protocol** **can** **be** **accessed,** **or** **state** **that** **a** **protocol** **was** **not** **prepared.**  Element:  • Indicate where the review protocol can be accessed (e.g. by providing a citation, DOI or link), or state that a protocol was not prepared. | 4 |  |  |
| **REGISTRATION** **AND** **PROTOCOL**  **(amendments)** | | **24c** | **Item:** **Describe** **and** **explain** **any** **amendments** **to** **information** **provided** **at** **registration** **or** **in** **the** **protocol.**  Element:  • Report details of any amendments to information provided at registration or in the protocol, noting: (a) the amendment itself; (b) the reason for the amendment; and (c) the stage of the review process at which the amendment was implemented. | 1 | This review has been registered in PROSPERO (registration number: CRD420251208327). After the registration was completed and during the first revision stage, we made timely and appropriate adjustments to the research plan based on the opinions of the reviewers. These adjustments mainly included the refinement of the data extraction form and the further definition of the disease spectrum range of the included literature. All these adjustments have been reflected in the methods section (see the "Data Items" and "Eligibility Criteria" subsections). The core research objective, search strategy, and main analysis method of the review remained unchanged after the adjustments. |  |
| **SUPPORT** | | **25** | **Item:** **Describe** **sources** **of** **financial** **or** **non-financial** **support** **for** **the** **review,** **and** **the** **role** **of** **the** **funders** **or** **sponsors** **in** **the** **review.**  Elements:  • Describe sources of financial or non-financial support for the review, specifying relevant grant ID numbers for each funder. If no specific financial or non- financial support was received, this should be stated.  • Describe the role of the funders or sponsors (or both) in the review. If funders or sponsors had no role in the review, this should be declared. | 19 |  |  |
| **COMPETING**  **INTERESTS** | | **26** | **Item:** **Declare** **any** **competing** **interests** **of** **review** **authors.**  Elements:  • Disclose any of the authors’ relationships or activities that readers could consider pertinent or to have influenced the review.  • If any authors had competing interests, report how they were managed for particular review processes. | 19 |  |  |
| **AVAILABILITY** **OF**  **DATA,** **CODE,** **AND** **OTHER** **MATERIALS** | | **27** | **Item:** **Report** **which** **of** **the** **following** **are** **publicly** **available** **and** **where** **they** **can** **be** **found:** **template** **data** **collection** **forms;** **data** **extracted** **from** **included** **studies;** **data** **used** **for** **all** **analyses;** **analytic** **code;** **any** **other** **materials** **used** **in** **the** **review.**  Elements:  • Report which of the following are publicly available: template data collection forms; data extracted from included studies; data used for all analyses;  analytic code; any other materials used in the review.  • If any of the above materials are publicly available, report where they can be found (e.g. provide a link to files deposited in a public repository).  • If data, analytic code, or other materials will be made available upon request, provide the contact details of the author responsible for sharing the materials and describe the circumstances under which such materials will be shared. | 19 |  |  |

*From:* Page MJ, McKenzie JE, Bossuyt PM, Boutron I, Hoffmann TC, Mulrow CD, et al. The PRISMA 2020 statement: an updated guideline for reporting systematic reviews. BMJ 2021;372:n71. doi: 10.1136/bmj.n71.

For more information, visit:<https://www.prisma-statement.org/>
