# Supplementary material for: Application of Large Language Models in Chronic Disease Care: Mixed Methods Systematic Review and Thematic Synthesis
Source: J Med Internet Res. 2026 Aug 11;28:e90744. doi: 10.2196/90744 (PMC13460802; doi:10.2196/90744)
Supplement: Supplementary file 5 [file jmir-v28-e90744-s005.docx]

The citation for the Synthesis Without Meta-analysis explanation and elaboration article is: Campbell M, McKenzie JE, Sowden A, Katikireddi SV, Brennan SE, Ellis S, Hartmann-Boyce J, Ryan R, Shepperd S, Thomas J, Welch V, Thomson H. Synthesis without meta-analysis (SWiM) in systematic reviews: reporting guideline BMJ 2020;368:l6890 <http://dx.doi.org/10.1136/bmj.l6890>

| **SWiM is intended to complement and be used as an extension to PRISMA** | | | |
| --- | --- | --- | --- |
| **SWiM reporting item** | **Item description** | **Page in manuscript where item is reported** | **Other*** |
| *Methods* | | | |
| **1** Grouping studies for synthesis | 1a) Provide a description of, and rationale for, the groups used in the synthesis (e.g., groupings of populations, interventions, outcomes, study design) | Page 4, 5 | Due to the heterogeneity in clinical and methodological aspects, no Meta-analysis was conducted. Instead, the thematic synthesis method was adopted, and the subjects were grouped based on the three dimensions of Orem's theory (the basic level, the intermediate level, and the top level). |
|  | 1b) Detail and provide rationale for any changes made subsequent to the protocol in the groups used in the synthesis | Page 4, 5 |  |
| **2** Describe the standardised metric and transformation methods used | Describe the standardised metric for each outcome. Explain why the metric(s) was chosen, and describe any methods used to transform the intervention effects, as reported in the study, to the standardised metric, citing any methodological guidance consulted | Page 4, 5 |  |
| **3** Describe the synthesis methods | Describe and justify the methods used to synthesise the effects for each outcome when it was not possible to undertake a meta-analysis of effect estimates | Page 4, 5 | Using the three-step thematic synthesis method by Thomas & Harden: line-by-line coding (NVivo 14, coder consistency 92.3%), formation of descriptive themes, and generation of analytical themes (mapped to Orem's three-dimensional framework). |
| **4** Criteria used to prioritise results for summary and synthesis | Where applicable, provide the criteria used, with supporting justification, to select the particular studies, or a particular study, for the main synthesis or to draw conclusions from the synthesis (e.g., based on study design, risk of bias assessments, directness in relation to the review question) | Page 4, 5 | Included were the studies rated as medium or high quality by MMAT; all 20 studies met the criteria. In the thematic synthesis, all studies were included equally without any additional prioritization. |
| **SWiM reporting item** | **Item description** | **Page in manuscript where item is reported** | **Other*** |
| **5** Investigation of heterogeneity in reported effects | State the method(s) used to examine heterogeneity in reported effects when it was not possible to undertake a meta-analysis of effect estimates and its extensions to investigate heterogeneity | Page 4, 5, 14, 15 | By constructing an evidence intensity area chart (Figure 6), the distribution of evidence for each study in the three-dimensional framework is visualized, and the areas of evidence concentration and absence are described in words. |
| **6** Certainty of evidence | Describe the methods used to assess certainty of the synthesis findings | Page 15, 16, 17 | Based on the MMAT quality rating and the evidence coverage of each dimension in the three-dimensional framework, a qualitative judgment is made. |
| **7** Data presentation methods | Describe the graphical and tabular methods used to present the effects (e.g., tables, forest plots, harvest plots).  Specify key study characteristics (e.g., study design, risk of bias) used to order the studies, in the text and any tables or graphs, clearly referencing the studies included | Page 8, 9, 10, 11, 12, 13, 14 | Table 1, Figures 2-6 |
| *Results* | | | |
| **8** Reporting results | For each comparison and outcome, provide a description of the synthesised findings, and the certainty of the findings. Describe the result in language that is consistent with the question the synthesis addresses, and indicate which studies contribute to the synthesis | Page 16-15 | Report on three topics separately. For each topic, cite specific studies and indicate the strength of the evidence. |
| *Discussion* |  |  |  |
| **9** Limitations of the synthesis | Report the limitations of the synthesis methods used and/or the groupings used in the synthesis, and how these affect the conclusions that can be drawn in relation to the original review question | Page 18 | It is pointed out that there are language limitations, potential publication bias, subjective synthesis bias, and limited cross-cultural applicability. However, these do not affect the direction of the main conclusion. |

PRISMA=Preferred Reporting Items for Systematic Reviews and Meta-Analyses.

*If the information is not provided in the systematic review, give details of where this information is available (e.g., protocol, other published papers (provide citation details), or website (provide the URL)).
